# Dysregulated Treg repair responses lead to chronic rejection after heart transplantation

**DOI:** 10.1172/JCI173593

**Published:** 2024-12-02

**Authors:** Jordan J.P. Warunek, Lu Fan, Xue Zhang, Sihua Wang, Steven M. Sanders, Tengfang Li, Lisa R. Mathews, Gaelen K. Dwyer, Michelle A. Wood-Trageser, Stephanie Traczek, Andrew Lesniak, Kassandra Baron, Hailey Spencer, Johnny Bou Saba, Emmanuel León Colón, Tracy Tabib, Robert Lafyatis, Mark A. Ross, Anthony J. Demetris, Simon C. Watkins, Steven A. Webber, Khodor I. Abou-Daya, Hēth R. Turnquist

**Affiliations:** 1 Thomas E. Starzl Transplantation Institute,; 2 Department of Surgery, and; 3 Department of Immunology, University of Pittsburgh School of Medicine, Pittsburgh, Pennsylvania, USA.; 4 School of Medicine, Tsinghua Medicine, Tsinghua University, Beijing, China.; 5 Department of Thoracic Surgery, Union Hospital, Tongji Medical College, Huazhong University of Science and Technology, Wuhan, China.; 6 Department of Kidney Transplantation, Center of Organ Transplantation, The Second Xiangya Hospital of Central South University, Changsha, China.; 7 Department of Pathology University of Pittsburgh School of Medicine, Pittsburgh, Pennsylvania, USA.; 8 Department of Infectious Disease and Microbiology, University of Pittsburgh School of Public Health, Pittsburgh, Pennsylvania, USA.; 9 Division of Rheumatology and Clinical Immunology, University of Pittsburgh School of Medicine, Pittsburgh, Pennsylvania, USA.; 10 Department of Cell Biology and; 11 Center for Biologic Imaging, University of Pittsburgh, Pittsburgh, Pennsylvania, USA.; 12 University of Arkansas for Medical Sciences, Little Rock, Arkansas, USA.; 13 McGowan Institute for Regenerative Medicine, University of Pittsburgh, Pittsburgh, Pennsylvania, USA.

**Keywords:** Immunology, Transplantation, Cellular immune response, Fibrosis, Organ transplantation

## Abstract

Chronic rejection (CR) after organ transplantation is alloimmune injury manifested by graft vascular remodeling and fibrosis that is resistant to immunosuppression. Single-cell RNA-Seq analysis of MHC class II–mismatched (MHCII-mismatched) heart transplants developing chronic rejection identified graft IL-33 as a stimulator of tissue repair pathways in infiltrating macrophages and Tregs. Using IL-33–deficient donor mice, we show that graft fibroblast–derived IL-33 potently induced amphiregulin (Areg) expression by recipient Tregs. The assessment of clinical samples also confirmed increased expression of Areg by intragraft Tregs also during rejection. Areg is an EGF secreted by multiple immune cells to shape immunomodulation and tissue repair. In particular, Areg is proposed to play a major role in Treg-mediated muscle, epithelium, and nerve repair. Assessment of recipient mice with Treg-specific deletion of Areg surprisingly uncovered that Treg secretion of Areg contributed to CR. Specifically, heart transplants from recipients with Areg-deficient Tregs showed less fibrosis, vasculopathy, and vessel-associated fibrotic niches populated by recipient T cells. Mechanistically, we show that Treg-secreted Areg functioned to increase fibroblast proliferation. In total, these studies identify how a dysregulated repair response involving interactions between IL-33^+^ fibroblasts in the allograft and recipient Tregs contributed to the progression of CR.

## Abstract 2

Local IL-33 triggers repair by infiltrating regulatory T cells (Tregs) but dysregulation of this Treg-mediated repair process contributes to fibrosis leading chronic transplant rejection.

## Graphical abstract

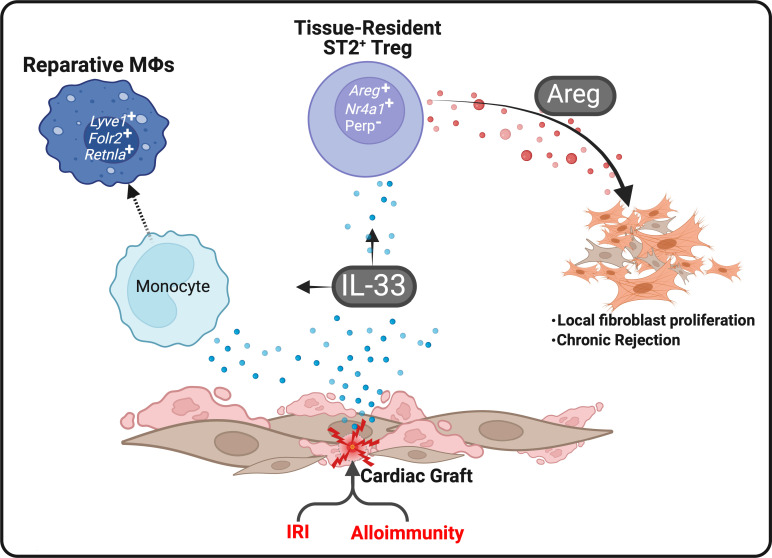

## Introduction

Solid organ transplants undergo initial damage from donor trauma, brain death, surgical manipulations, and ischemia-reperfusion injury (IRI). Throughout the life of the transplant, the recipient’s immune responses to alloantigens (AlloAgs) will also cause damage (1). Injuries release self-derived molecules containing damage-associated molecular patterns (DAMPs) that alert immune cells and shape their functions at the injury site. Proinflammatory DAMPs, such as HMGB1 or vimentin, along with proinflammatory cytokines, initiate local inflammation by recruiting and activating neutrophils, monocytes, and macrophages (2). Proinflammatory stimuli may also mediate innate immune cell “training” to amplify macrophage responses (3). The early generation of proinflammatory macrophages following transplantation is linked to adverse outcomes, and targeting proinflammatory DAMPs or related pathways has shown efficacy in experimental models (3, 4). Reducing ischemia times to decrease IRI reduces early rejection and the subsequent development of chronic allograft vasculopathy (CAV) and fibrosis leading to chronic rejection (CR) (5–8). Decreasing CR is an urgent need, as almost 50% of heart transplants develop CAV after 10 years, and CR is a leading cause of death after the first post-transplantation year (8).

Cardiac injury studies have also revealed a beneficial role for neutrophils and macrophages in repair responses. These cells collaborate to clear dead and damaged tissues, as well as generate or act as repair signals for other immune cells (2, 9, 10). Once initiated, the tissue repair process is completed in orchestrated resolution and repair phases that are directed by reparative DAMPs, resolvins, lipoxins, maresins, and type 2 cytokines (2, 9, 10). These signals support the generation of reparative macrophages that suppress local innate and adaptive immune responses, direct revascularization, and attract and differentiate tissue fibroblasts into myofibroblasts that modulate the local extracellular matrix (2, 9, 10). Interestingly, Tregs have shown distinct reparative abilities separate from their suppressive functions following lung, heart, and muscle injuries (11–14). Specifically, Tregs’ recognition of the DAMP, IL-33 via the IL-33 receptor stimulation 2 (ST2) initiates several distinct reparative actions. Tregs that express ST2 (ST2^+^ Tregs), while suppressive, also foster tissue repair and restore tissue function through the IL-33–induced secretion of amphiregulin (Areg). Areg is a member of the EGF family, which causes stem cell proliferation and differentiation through actions on the EGFR (15). IL-33 also stimulates IL-13 secretion by ST2^+^ Tregs, which act to generate arginase 1 (Arg1^+^) macrophages that resolve local inflammation and initiate repair after lung injury (14). Yet, allograft transplantation causes a unique immunological situation in which non-self, allogeneic signals may profoundly alter the response of recipient immune cells tasked with tissue repair. Interestingly, Areg has been implicated in chronic lung allograft dysfunction by increasing hyaluronan and hyaluronan synthase expression in bronchial epithelial cells (16). How Treg-mediated repair pathways shape heart transplantation (HTx) outcomes deserves focused and thorough investigation.

Using IL-33 as a model injury signal in mouse HTx studies, we discovered that IL-33 acts as a reparative-type DAMP that locally triggers response pathways associated with tissue repair in infiltrating recipient macrophages and Tregs in the graft. Single-cell RNA-Seq (scRNA-Seq) of HTx isolates revealed that IL-33–stimulated Treg were the dominant source of Areg in the transplant in both mice and humans. Treg-produced Areg directly promoted fibroblast proliferation, and recipients with Areg-deficient Tregs had reduced perivascular fibrosis and T cell infiltrate. Thus, we show that recipient repair responses become dysregulated in the allograft and presumed beneficial immune cells like Tregs can contribute to CR.

## Results

### Utilizing scRNA-Seq to define the immune cells shaping CR following transplantation.

**Figure 1:**
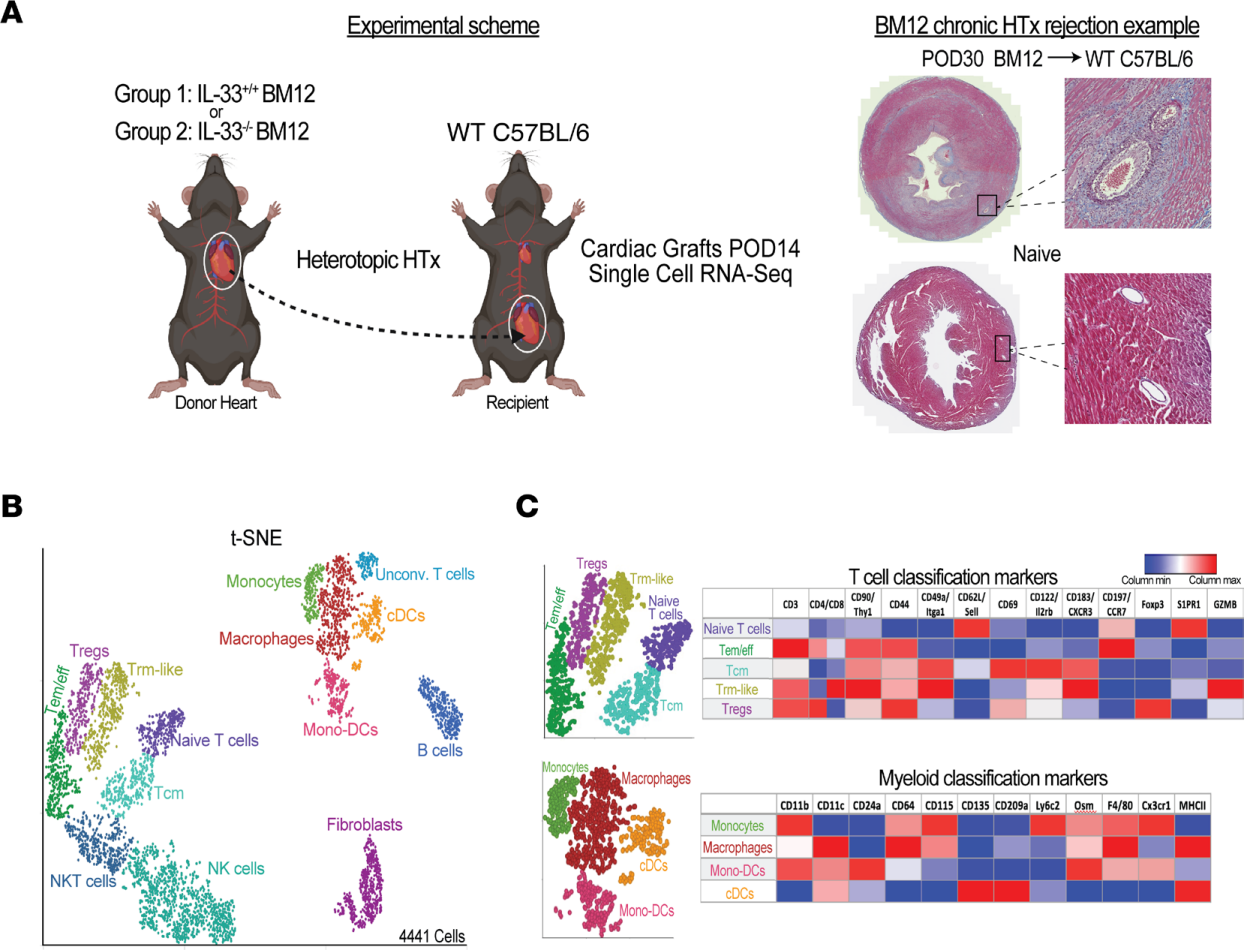
Effect of graft IL-33 on the immune cell landscapes following HTx. (**A**) Experimental schematic illustrating the use of scRNA-Seq to analyze *Il33^+/+^* or *Il33^–/–^* Bm12 heart grafts at POD14 following heterotopic HTx into B6 recipients (*n* = 3/group). Created with BioRender.com. (**B**) *t*-SNE projection of graft immune cell populations (*n* = 4,441 cells). Unconv., unconventional. (**C**) Lineage analysis to identify T cell and myeloid clusters (*n* = 3 hearts/group).

The immune cells and mechanisms involved in CR after HTx remain poorly understood. To study how injury signals affect the graft immune compartment after HTx, we performed scRNA-Seq comparing cell isolates from IL-33^+^ and IL-33^–^ Bm12 heart allografts transplanted into C57BL/6 (B6) recipients at postoperative day (POD) 14. Bm12 mice have a mutation that generates I-A^Bm12^, an altered I-A molecule that is recognized by B6 CD4^+^ T cells to cause CR-associated fibrosis and vasculopathy within 30 days of HTx (Figure 1A) (17, 18). We analyzed a total of 4,441 cells (*n* = 3 hearts/group), with 2,762 cells in IL-33^+^ grafts and 1,679 cells in IL-33^–^ grafts (Supplemental Figure 1A; supplemental material available online with this article; https://doi.org/10.1172/JCI173593DS1). Dimensionality reduction using *t*-distributed stochastic neighbor embedding (t-SNE) projected a heterogenous landscape of immune cell neighborhoods. Graph-based clustering validated the distinct nature of these neighborhoods; separate neighborhoods coincided with separate clusters (Supplemental Figure 1, B–D). Clusters/neighborhoods were annotated on the basis of differential expression of canonical markers, which resolved populations of lymphocytes that included NK cells, NK T (NKT) cells, B cells, and subtypes of CD4^+^ Th cells including naive T (Tn) cells, effector/effector memory T (Teff/Tem) cells, central memory T (Tcm) cells, resident memory-like T (Trm-like) cells, and Tregs (Figure 1, B and C). The myeloid compartment was composed of monocytes, macrophages, conventional DCs (cDCs), and monocyte-derived DCs (mono-DCs) (Figure 1, B and C). The complete expression of markers used to profile myeloid and T cells are detailed in Supplemental Figure 1, B–D. Our scRNA-Seq analysis provided a comprehensive transcriptomic landscape of myeloid and adaptive immune cells in early post-HTx grafts.

### Cardiac IL-33 stimulates the generation of reparative macrophages.

**Figure 2:**
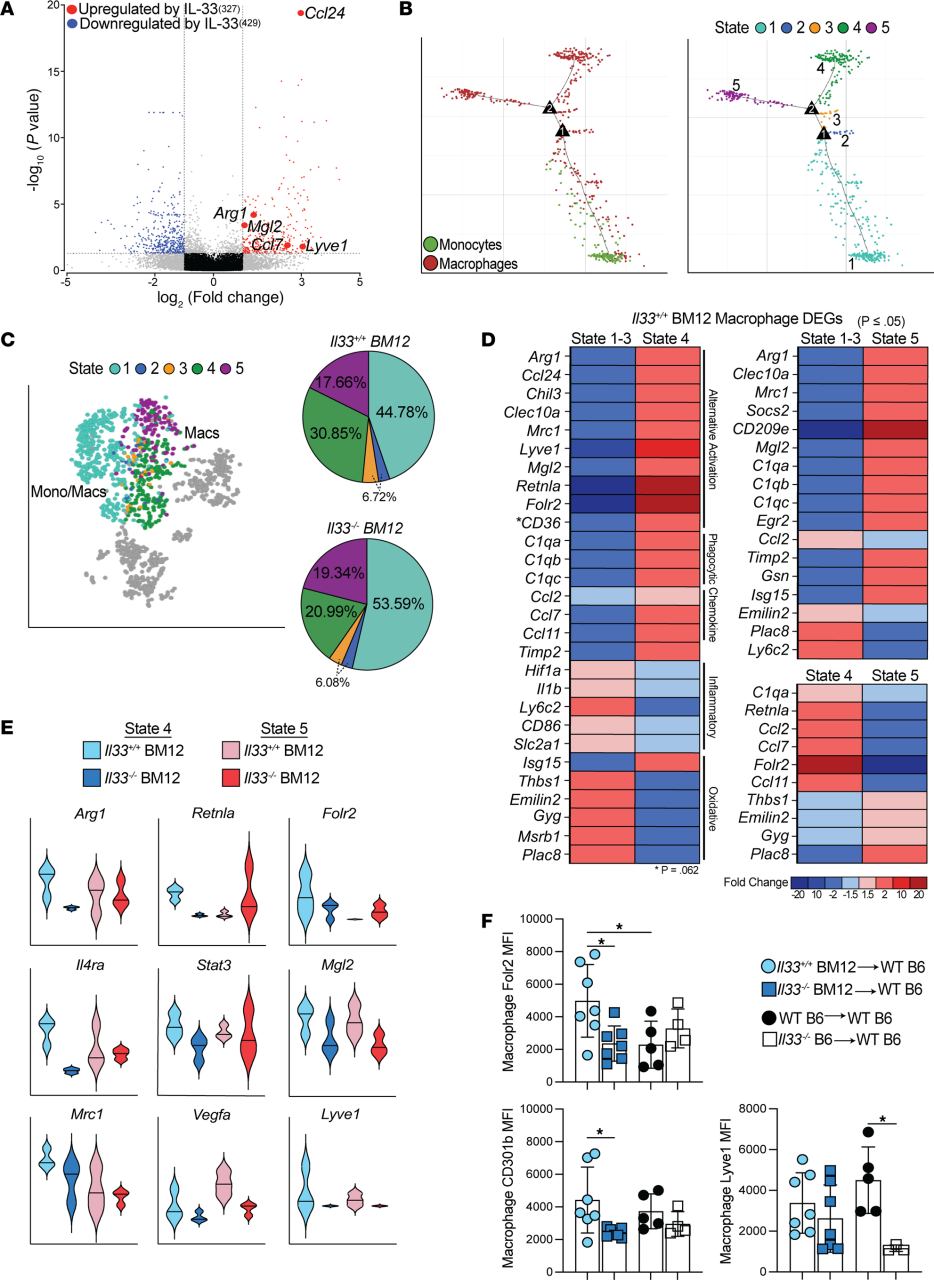
Local cardiac IL-33 orchestrates reparative macrophage differentiation. (**A**–**E**) scRNA-Seq at POD14 examining monocytes and macrophages in donor *Il33^+/+^* or *Il33^–/–^* Bm12 hearts transplanted into B6 mice. (**A**) Volcano plot comparison of differentially expressed macrophage genes. Graft IL-33 significantly upregulated 327 genes, while downregulating 429 genes. (**B**) Single-cell pseudotime trajectory of monocyte and macrophage Monocle states in *Il33^+/+^* Bm12 grafts. (**C**) Distribution of Monocle states in *Il33^+/+^* and *Il33^–/–^* Bm12 grafts. (**D**) Heatmap comparing differential gene expression of the specified Monocle states in *Il33^+/+^* Bm12 grafts. Mono, monocytes; Macs, macrophages. (**E**) Comparison of state 4 and state 5 genes in *Il33^+/+^* and *Il33^–/–^* Bm12 grafts (*n* = 3 hearts/group). (**F**) MFI of the indicated proteins for recipient macrophages (CD45.1^+^CD45 i.v.^–^, CD3^–^CD11b^+^Ly6G^–^CD11c^–^F4/80^+^) in *Il33^+/+^* and *Il33^–/–^* Bm12 or *Il33^+/+^* and *Il33^–/–^* syngeneic grafts at POD14 (*n* = 7 for Bm12 grafts; *n* = 3–5 for syngeneic grafts). The results represent cumulative data from 6 independent experiments. Individual data points are depicted in the graphs, along with the group mean ± SD. *P ≤ 0.05, by 1-way ANOVA with Tukey’s multiple-comparison test.

The absence of IL-33 in the graft resulted in significant changes in gene expression across all cell populations analyzed (Supplemental Data File 1). We have previously demonstrated the role of local IL-33 in limiting CCR2^+^ monocyte differentiation into proinflammatory macrophages at HTx POD3 (19). At HTx POD14, the absence of IL-33 in the graft altered the gene expression profiles of recipient macrophages. We identified 756 differentially expressed genes (DEGs) between IL-33^+^ and IL-33^–^ Bm12 HTx macrophages (≥|2-fold|, *P* ≤ 0.05) (Supplemental Data File 1). IL-33 was found to contribute positively to the generation of reparative-type macrophages expressing *Ccl24*, *Arg1*, *Mgl2*, and *Lyve1* (Figure 2A). Using pseudotime trajectory inference, we examined the effect of local IL-33 on monocyte-to-macrophage differentiation following HTx. We identified 5 differentiation states with 2 bifurcation points in their cell-fate development (Figure 2, B–D). Comparing the abundance of these states between IL-33^+^ and IL-33^–^ Bm12 grafts revealed that IL-33 restrained state 1 monocytes and macrophages, while favoring the generation of state 4 macrophages (Figure 2C). The transcriptional profiles of these states in IL-33^+^ as compared with IL-33^–^ hearts, which we report as fold changes of mean expression, indicated that the abundant state 1 population, along with minor contributions from states 2 and 3, were composed of monocytes (*Plac8* and *Ly6c2*) (20) and proinflammatory and oxidative macrophages (*Il1b*, *Cd86*, and *Hif1a*) (21, 22) (Figure 2D). Conversely, transcriptionally reparative-type macrophages occupied states 4 and 5 and shared expression of the markers ascribed to alternative macrophage activation (22) including *Arg1*, *Clec10a*, *Mrc1*, and *Mgl2* (Figure 2D). State 4 and 5 macrophages were also enriched for transcripts assigned to phagocytic reparative subsets (22) with upregulated complement component C1q genes (*C1qa*, *C1qb*, *C1qc*) (Figure 2D) that aid the removal of apoptotic cells during the healing process (22). The robust presence of reparative macrophages is counterintuitive, given the availability of proinflammatory DAMPs (3, 4) and a local alloreactive CD4^+^ immune response (4, 18). Thus, potent reparative stimuli like IL-33 also appear highly influential in the transplant microenvironment.

Monocyte-derived macrophages upregulate *Retnla* as they adopt tissue residency (22), and *Folr2* expression is characteristic of donor cardiac graft–resident macrophages (23). *Retnla* and *Folr2*, as well as *Arg1*, were uniquely upregulated in state 4 macrophages, and this was dependent on local IL-33 (Figure 2D). The presence of IL-33 in the graft markedly increased state 4 and 5 macrophage expression of other genes indicative of reparative macrophages (*Mrc1*, *Il4ra*, *Lyve1*, *Ccl24*, and *Vegfa*) (Figure 2E). At HTx POD14, macrophage Folr2 and CD301b (encoded by *Mgl2*) protein expression was similarly modulated by IL-33 in allografts, where Lyve1 was most influenced by IL-33 in the absence of alloimmune-mediated injury (Figure 2F and Supplemental Figure 2). Thus, IL-33 appeared to act early in the differentiation process, supporting 2 cell population states of reparative macrophages. These observations suggest that transplant surgery and immune cell injury to the graft released IL-33, which worked with local signals to help coordinate the differentiation of reparative macrophages from infiltrating monocytes and macrophages.

### Local IL-33 programs Tregs for tissue residency and injury repair.

**Figure 3:**
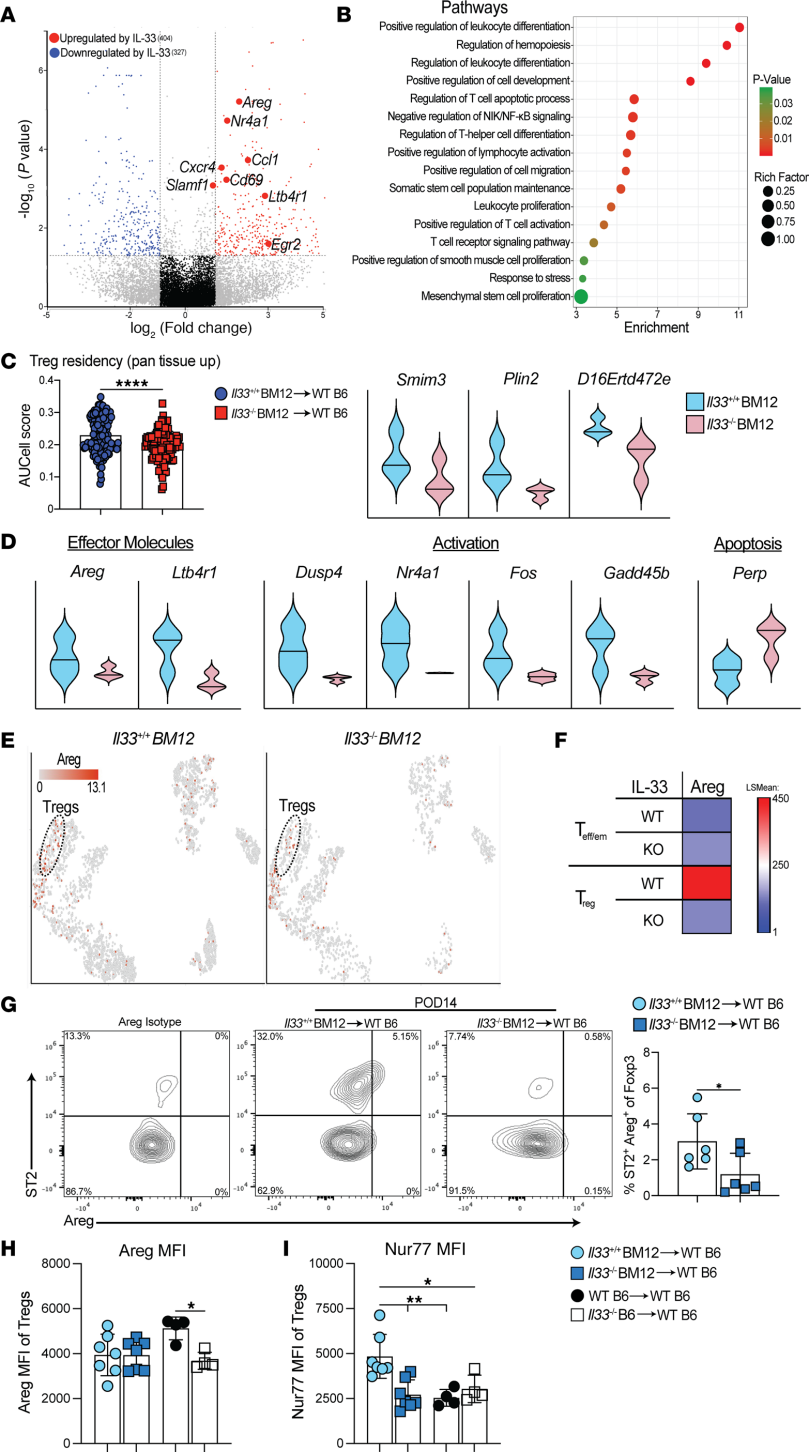
IL-33 promotes tissue-resident Tregs and enhances tissue repair programs. (**A**–**F**) scRNA-Seq analysis of Tregs in Bm12 grafts at POD14 comparing *Il33^+/+^* and *II33^–/–^* Bm12 grafts. (**A**) Volcano plot depicting DEGs in Tregs. (**B**) Pathways modulated by IL-33. (**C**) IL-33 contribution to the published Treg pan-tissue signature (28) and (**D**) selected genes significantly modulated by IL-33 in *Il33^+/+^* versus *II33^–/–^* Bm12 grafts. (**C**) ****P ≤ 0.0001, by Mann-Whitney *U* test. up, upregulated. (**E**) *t*-SNE projection of Areg-expressing cells. (**F**) Heatmap comparing Areg expression in *Il33^+/+^* versus *Il33^–/–^* in conventional T cells and Tregs (*n* = 3 hearts/group). LSMean, least squares mean. (**G**) Representative flow cytometry plots and frequency quantification of ST2^+^Areg^+^ Tregs (CD45.1^+^CD45 i.v.^–^, CD3^+^CD4^+^CD8^–^Foxp3^+^) in *Il33^+/+^* and *II33^–/–^* Bm12 grafts. (**H**) MFI of Areg and (**I**) Nurr77 expression in intragraft Tregs at POD14. (**G**–**I**) *n* = 7 for Bm12 grafts; *n* = 4 for syngeneic grafts. *P ≤ 0.05 and **P ≤ 0.01, by 1-way ANOVA with Tukey’s multiple-comparison test. The results represent cumulative data from 6 independent experiments. Individual data points are depicted in the graphs, along with the group mean ± SD.

We also observed that 731 genes were significantly modulated (≥|2-fold|, *P* ≤ 0.05) in Tregs by local IL-33 (Figure 3A and Supplemental Data Files 1 and 2). These included genes regulating T cell differentiation, activation, function, and survival (Figure 3B). Tregs found in nonlymphoid tissues, also referred to as “tissue Tregs,” undergo stepwise differentiation and specialization initiated in secondary lymphoid organs (SLOs) but then are further instructed and maintained by unknown tissue-specific stimuli (24–27). Transcriptomics data from Tregs in visceral adipose tissue, muscle, skin, colonic lamina propria, and brain have been used to define the transcriptional features of pan–tissue-resident Tregs (28). While IL-33 signaling is believed to play a crucial role in the development and maintenance of tissue-resident Tregs (28), the extent to which ST2 stimulation modifies the Treg transcriptome in tissues compared with its role in early differentiation in SLOs has remained unclear. Using the unique aspects of rodent transplantation models, in which an IL-33^–^ graft is transplanted into a WT recipient, we established that tissue-derived IL-33 was indeed a dominant driver of the pan-tissue Treg transcriptome (28) (Figure 3C and Supplemental Data File 3). Local IL-33 induced a range of genes associated with Treg tissue residency (28), including effector molecules (*Ltb4r1* and *Areg*) and antiapoptotic genes (Figure 3D). On the basis of these data generated using a mouse transplant model, we demonstrated that peripheral tissue IL-33 rapidly induced a transcriptional profile characteristic of tissue-resident Tregs in graft-infiltrating Tregs.

The scRNA-Seq data also revealed that IL-33 stimulation in grafts enhanced the expression of *Il1rl1* (also known as *St2*), along with *Areg* and *il13* in Tregs (Figure 3A and Supplemental Data File 1), with both Areg and IL-13 implicated in ST2^+^ Treg reparative functions after lung and muscle injury (12–14). Areg can be secreted by numerous immune cells (15), yet it was clear that IL-33–stimulated Tregs were the dominant Areg^+^ immune cell in IL-33^+^ heart grafts (Figure 3, E and F). IL-33 also augmented Treg expression of T cell receptor (TCR) response transcripts, such as *Egr2*, *Nr4a1*, *Cd69*, *Ccl1*, and *Slamf1* (Figure 3A and Supplemental Data File 1). This is reminiscent of our demonstration that IL-33 from fibroblast reticular cells (FRCs) in the SLOs acts as a costimulatory molecule that augments CD4^+^ TCR signaling (29). Consistent with the above-described scRNA-Seq data, intragraft Tregs showed a significant reduction in the frequency of ST2^+^ and Areg^+^ cells in the absence of local IL-33 in both allogeneic (Figure 3G) and syngeneic (Supplemental Figures 2 and 3) grafts. The level of Areg expression, however, was only significantly increased in Tregs from syngeneic HTx (Figure 3H). Protein levels of Nur77 (encoded by *Nr4a1*) in Tregs were only amplified in allografts (Figure 3I), suggesting that IL-33 augmented alloreactive TCR signaling. The IL-33–mediated gene modulations in Tregs resulted in significant enrichment scores (*P* ≤ 0.05) in gene sets associated with somatic stem cell maintenance, hemopoiesis, cell migration, cell development, smooth muscle, mesenchymal cell proliferation, as well as leukocyte differentiation, activation, and apoptosis (Figure 3B and Supplemental Data File 2). Clearly, IL-33 released from injured stromal cells profoundly influenced the immunobiology of Tregs in the local environment of heart grafts.

### Treg-derived Areg supports vascular occlusion and perivascular fibrosis.

**Figure 4:**
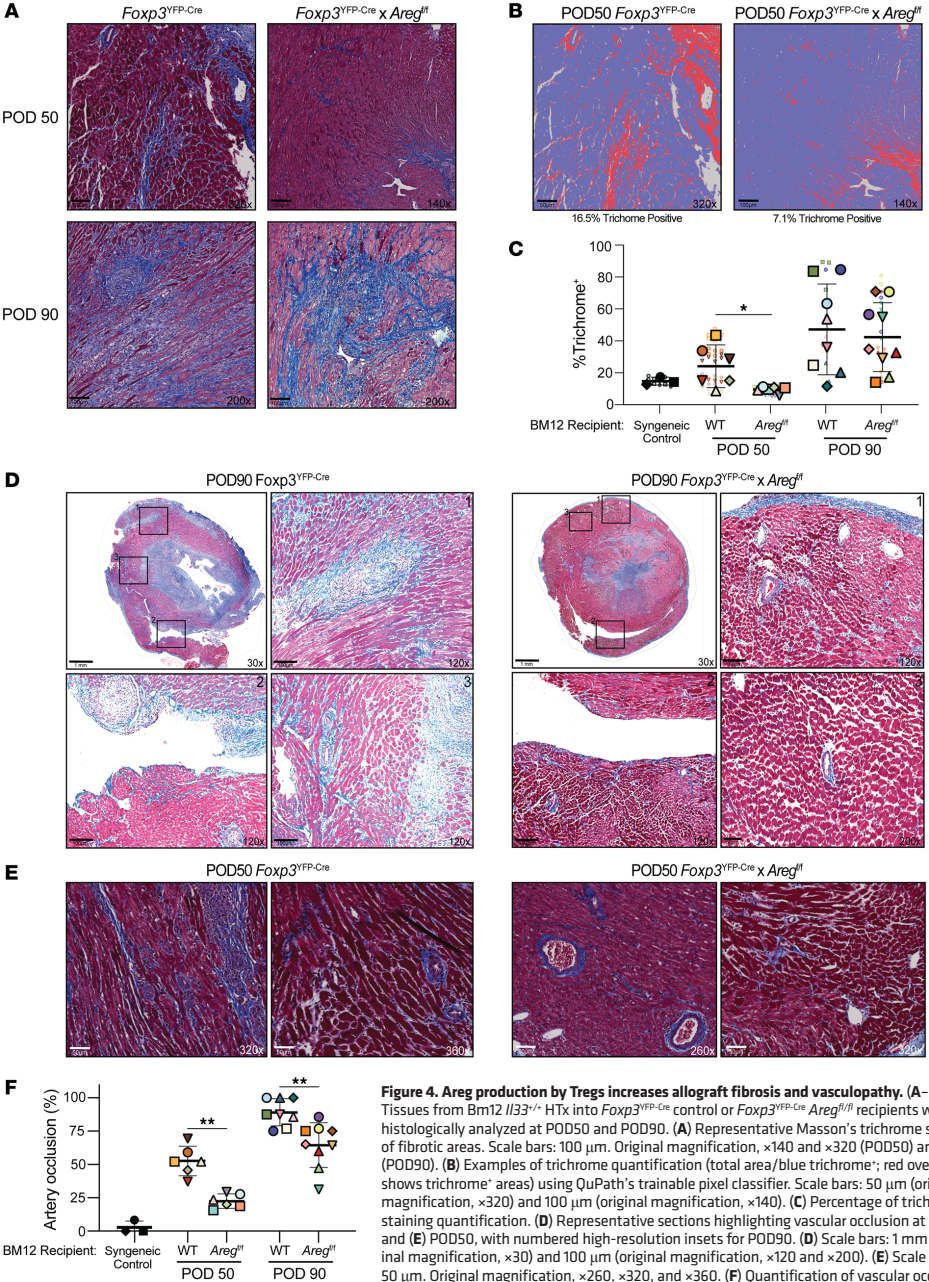
Areg production by Tregs increases allograft fibrosis and vasculopathy. (**A**–**F**) Tissues from Bm12 *Il33^+/+^* HTx into *Foxp3*^YFP-Cre^ control or *Foxp3*^YFP-Cre^ *Areg^fl/fl^* recipients was histologically analyzed at POD50 and POD90. (**A**) Representative Masson’s trichrome staining of fibrotic areas. Scale bars: 100 μm. Original magnification, ×140 and ×320 (POD50) and ×200 (POD90). (**B**) Examples of trichrome quantification (total area/blue trichrome^+^; red overlay shows trichrome^+^ areas) using QuPath’s trainable pixel classifier. Scale bars: 50 μm (original magnification, ×320) and 100 μm (original magnification, ×140). (**C**) Percentage of trichrome^+^ staining quantification. (**D**) Representative sections highlighting vascular occlusion at POD90 and (**E**) POD50, with numbered high-resolution insets for POD90. (**D**) Scale bars: 1 mm (original magnification, ×30) and 100 μm (original magnification, ×120 and ×200). (**E**) Scale bars: 50 μm. Original magnification, ×260, ×320, and ×360. (**F**) Quantification of vascular occlusion (total vessels/occluded vessels). For **C** and **F**, 3 syngeneic and 6–9 BM12 grafts/group were evaluated, and 1 or 2 depths/graft were quantitated. Cumulative data from 6 independent experiments are shown. Large symbols represent the means from individual grafts, and color- and (**C**) symbol-matched small symbols provide the values for each sample section. The thick bar and error bars represent the mean ± SD calculated from the biological sample means. *P ≤ 0.05 and **P ≤ 0.01, by Mann-Whitney *U* test.

Treg secretion of Areg is instrumental to early tissue injury repair after muscle, lung, and neuronal injury (11–13, 30). To define how Treg-derived Areg shapes graft repair and transplantation outcomes, we performed heterotopic transplantation of *Il33^+/+^* Bm12 hearts into B6 *Foxp3*^YFP-Cre^ or *Foxp3*^YFP-Cre^ *Areg^fl/fl^* mice that had profoundly ablated Areg secretion in response to IL-33 in vitro and in vivo (Supplemental Figure 4). At HTx POD50 and POD90–100, heart grafts were examined for fibrosis and vasculopathy. Using the bioimage analysis program QuPath to calculate the percentage of fibrosis, we observed an unexpected, but significant, decrease in overall fibrosis in Bm12 grafts in *Foxp3*^YFP-Cre^ *Areg^fl/fl^* recipients versus those in *Foxp3*^YFP-Cre^ mice at POD50 (Figure 4, A–C). Relatedly, *Foxp3*^YFP-Cre^ control grafts showed a significant increase in vascular occlusion at both time points (Figure 4, D–F). However, we found no differences in CD4^+^ or CD8^+^ T cell activation, memory, or differentiation in *Foxp3*^YFP-Cre^ *Areg^fl/fl^* Tregs recipients of Bm12 allografts at POD90 when compared with *Foxp3*^YFP-Cre^ controls (Supplemental Figure 5). These data establish that an effector mechanism used by Tregs for tissue repair became dysregulated after HTx to exacerbate fibrosis and vasculopathy, culminating in CR.

### Treg-promoted perivascular fibrosis provides a niche in the graft for infiltrating CD3^+^ T cells.

**Figure 5:**
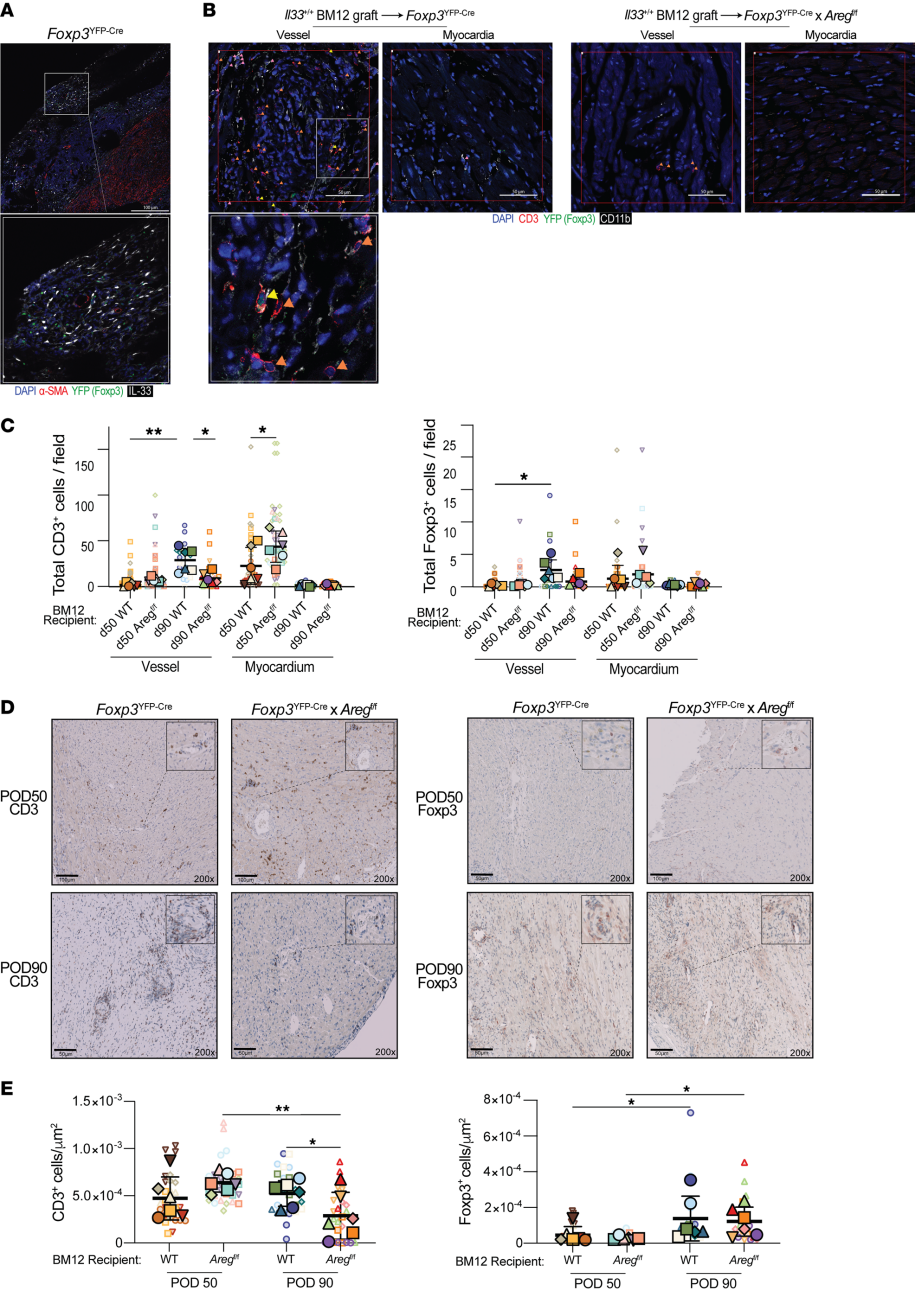
Treg-derived Areg promotes T cell accumulation around graft vessels. (**A**–**E**) Bm12 grafts were harvested after POD50 or POD90 from *Foxp3*^YFP-Cre^ or *Foxp3*^YFP-Cre^ *Areg^fl/fl^* recipient mice. Graphs show quantitative analysis of CD3^+^ T cells and CD3^+^Foxp3^+^ Tregs. (**A**) Representative POD90 Bm12 heart grafts in *Foxp3*^YFP-Cre^ recipients stained for IL-33 (white), α-SMA (red), Foxp3 (green), and DAPI (blue). Enlarged inset indicates high-resolution area (original magnification, ×110). Scale bar: 100 μm. (**B**) Representative POD90 Bm12 graft stained for CD3 (red), Foxp3 (green), CD11b (white) and DAPI. Enlarged inset indicates high-resolution area. Scale bars: 50 μm. (**C**) Quantification of CD3^+^ or Foxp3^+^ cells in myocardial or blood vessel areas. (**D**) IHC detection of CD3^+^ or Foxp3^+^ cells across the entire transplant sections. Scale bars: 50 μm and 100 μm. Insets in **D** present higher magnification of the indicated areas. (**E**) Quantification of CD3^+^ or Foxp3^+^ cells across the entire transplant sections was normalized to the total area (*n* = 6–9/group). In these studies, 6 BM12 grafts/group were evaluated, and 2 depths/graft were quantitated. Large symbols in **C** and **E** indicate the mean counts from individual grafts and color- and symbol-matched small icons provide the values for each sample section. The thick bar and error bars represent the mean ± SD calculated from the biological sample means. *P ≤ 0.05 and **P ≤ 0.01, by 1-way ANOVA with Tukey’s multiple-comparison test.

Perivascular cuffing, in which T cells and macrophages surround graft vasculature, is a histological feature of chronically rejecting heart transplants (31, 32). Persistent local alloimmune responses are believed to trigger intimal thickening by increasing vascular smooth muscle cells and extracellular matrix synthesis. We quantified CD3^+^, CD11b^+^, and Foxp3^+^ cells in the myocardia or within 100 μm of the vessel adventitia by immunohistochemistry (IHC) and immunofluorescence (IF) to understand how Areg-induced perivascular fibrotic areas altered immune infiltration and graft residency at POD50 and POD90–100 (Figure 5). At POD50, we detected a significant increase of CD3^+^ cells in the myocardium of grafts from *Foxp3*^YFP-Cre^ *Areg^fl/fl^* recipient mice (Figure 5, A–C). However, the CD3^+^ infiltrate in the graft myocardium cleared by POD90 in both *Foxp3*^YFP-Cre^ and *Foxp3*^YFP-Cre^ *Areg^fl/fl^* recipients, with the CD3^+^ infiltration now predominately confined near blood vessels of *Foxp3*^YFP-Cre^ mice (Figure 5, A–C). Conversely, grafts from *Foxp3*^YFP-Cre^ *Areg^fl/fl^* mice displayed a profound reduction in CD3^+^ cells near the vasculature, but a similar number of Tregs (Figure 5, A–C). Immunohistochemical analysis confirmed that, while *Foxp3*^YFP-Cre^ and *Foxp3*^YFP-Cre^ *Areg^fl/fl^* had similar numbers of Tregs (Figure 5, D and E), Treg-derived Areg in *Foxp3*^YFP-Cre^ recipients was again associated with a significant increase in overall CD3^+^ T cells and CD11b^+^ cells at POD90 near the vasculature at POD90 (Figure 5E and Supplemental Figure 6).

It has been reported that Areg may be important to Treg-suppressive capacity (33, 34), thus we conducted an in vitro Treg suppression assay to determine whether Treg-produced Areg played any role in limiting CD8^+^ or CD4^+^ T cell proliferation. There were, however, no differences observed in the ex vivo suppressive activity between splenic *Foxp3*^YFP-Cre^ versus *Foxp3*^YFP-Cre^ *Areg^fl/fl^* Tregs (Supplemental Figure 4C). Attempts to isolate sufficient numbers of intragraft Treg to assess their suppressive capacity proved technically nonviable. Yet, the data above argue against decreased local Treg-suppressive function mediating the observed phenotype, as we saw improvements in outcomes and a reduced presence of CD3^+^ cells at later time points. If Areg was needed for local suppression in the graft, one would expect to see the opposite (i.e., increased CD3^+^ T cells in local niches in recipients with Areg-deficient Tregs and less vasculopathy). Instead, these data fit a scenario in which Treg production of Areg contributes to increased vasculopathy and fibrosis, particularly around the vasculature where immune cells consolidate over time.

### Intragraft Treg expression of Areg is increased during clinical rejection.

**Figure 6:**
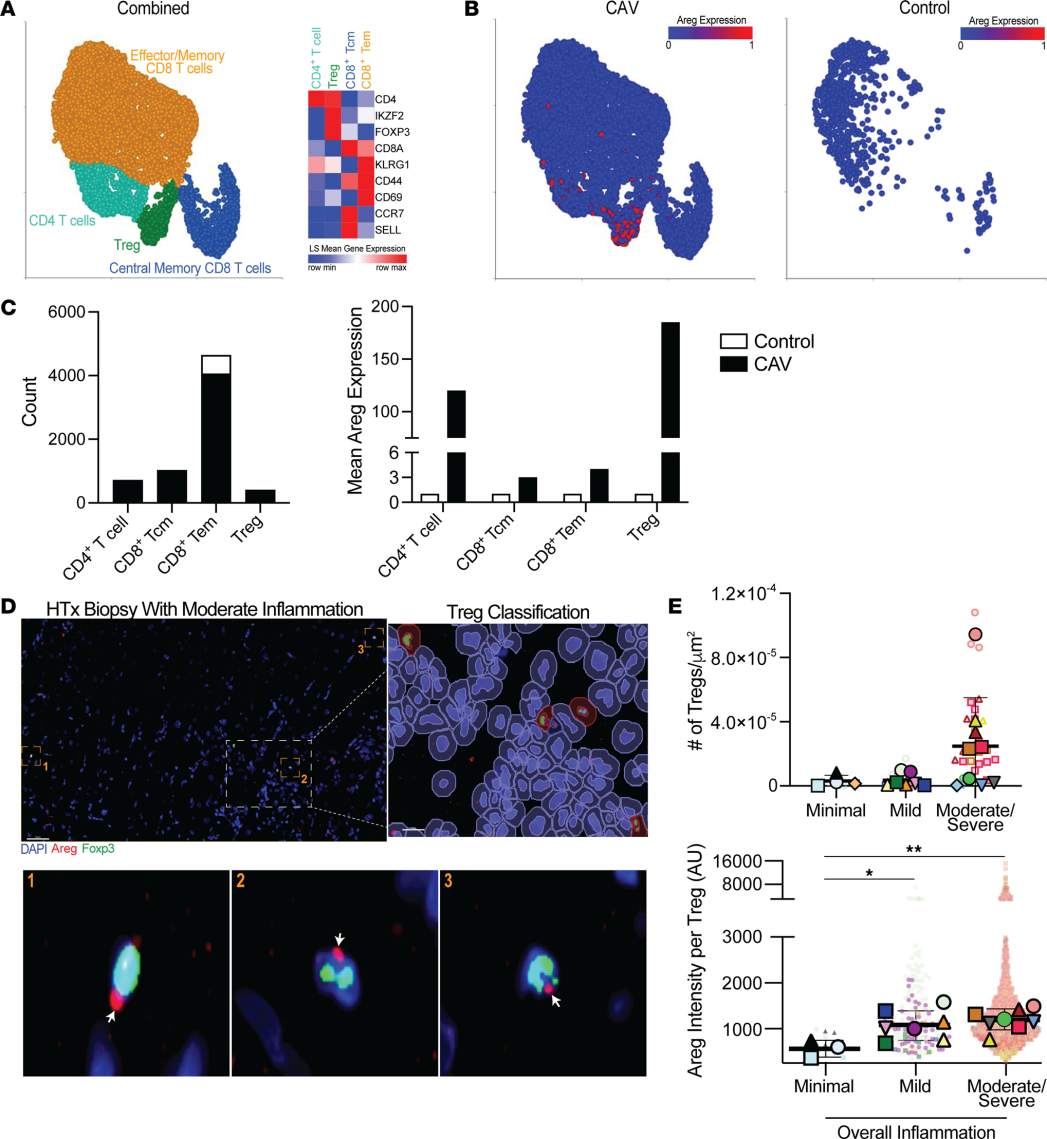
Augmented Areg in Treg is observed during clinical graft rejection and chronic allograft vasculopathy. (**A**–**C**) snRNA-Seq of heart samples from patients with severe CAV versus controls (GSE203548). (**A**) UMAP projection of all samples with T cell classifications labeled and highlighted by color. Heatmap depicts the expression of lineage markers used to assign neighborhoods. (**B**) Nuclei from the CAV samples or control samples were separated in the UMAP projection, respectively. (**C**) Counts of the T cell types in CAV and control samples and mean expression of Areg in the T cell types. (**D** and **E**) Assessment of EMBs for Areg (red), Foxp3 (green), and DAPI (blue). (**D**) Representative immunofluorescence images of an EMB displaying QuPath trainable cell detection to identify Tregs and Areg^+^ Tregs. White arrows indicate Areg staining. Scale bars: 50 μm. Insets present higher magnification of the indicated areas (original magnification, ~×2,000). (**E**) Number of Tregs and their level of Areg expression grouped by EMBs with minimal, mild, and moderate/severe allograft inflammation (*n* = 3–4 minimal, 7 = mild, 8 = moderate/severe). Large symbols depict the mean cell counts for individual patients, and color- and symbol-matched small symbols provide the cell values for each sample section. The thick bar and error bars represent the mean ± SD calculated from the individual patient means. *P ≤ 0.05 and **P ≤ 0.01, by 1-way ANOVA with Tukey’s multiple-comparison test.

To gain insights into whether Treg production of Areg may contribute to CR clinically after HTx, we analyzed publicly available single-nucleus RNA-Seq (snRNA-Seq) data on tissue samples from 4 individuals with severe CAV at the time of retransplantation and endomyocardial biopsy (EMB) specimens from 3 individuals after transplantation without CAV (Gene Expression Omnibus [GEO] accession number: GSE203548) (35). To investigate T cells in this dataset, we selected the cluster that expressed protein tyrosine phosphatase receptor type C (*Ptprc*), which encodes CD45, CD5, and CD3E/-G/-D (CD3). We then utilized uniform manifold approximation and projection (UMAP) to project the T cells in lower dimensional space on the basis of their gene expression profile. T cell neighborhoods were classified according to their differential expression of CD4, CD8, FOXP3, IKZF2, CCR7, CD44, SELL (also known as CD62L), CD69, and KLRG1. This distinguished neighborhoods of CD8^+^ T cells that included Tcm and Tem cells. In addition, populations of CD4^+^ T cells were evident, as well as Tregs (Figure 6A). Splitting the UMAP projections on the basis of whether the samples were from CAV or control (Figure 6B) tissues demonstrated that Tregs were only present in CAV samples (Figure 6, B and C). When we matched data from our mouse CAV model, we found that *Areg* gene expression was highest in Tregs (Figure 6C). We next assessed EMBs from another cohort of pediatric heart transplant recipients at times of pathologist-classified mild, minimal, and moderate/severe overall grading of inflammation, which typically correlated with a diagnosis of acute cellular rejection (International Society for Heart and Lung Transplantation grade ≥2R; see Supplemental Table 1 for specific details) after the first year using multiplex IHC for Areg and Foxp3 (Figure 6, D and E). QuPath-based calculation of the number of Tregs demonstrated a trend toward the expected (36) increase of Foxp3^+^ cells with increasing severity of inflammation/rejection (Figure 6E). Areg^+^Foxp3^+^ cells were common in the moderate and severe inflammation samples (Figure 6E), with significantly increased intensity of Areg measures being observed in Tregs in EMB at times of mild and moderate/severe inflammation (Figure 6E) and notably in explanted heart transplants failing due to CAV (Supplemental Figure 7A-B). In total, these data support the observation that the mechanisms identified in the mouse model of Bm12 allograft transplantation into B6 recipients are active in clinical cardiac transplantation.

### Treg-secreted Areg promotes fibroblast proliferation.

**Figure 7:**
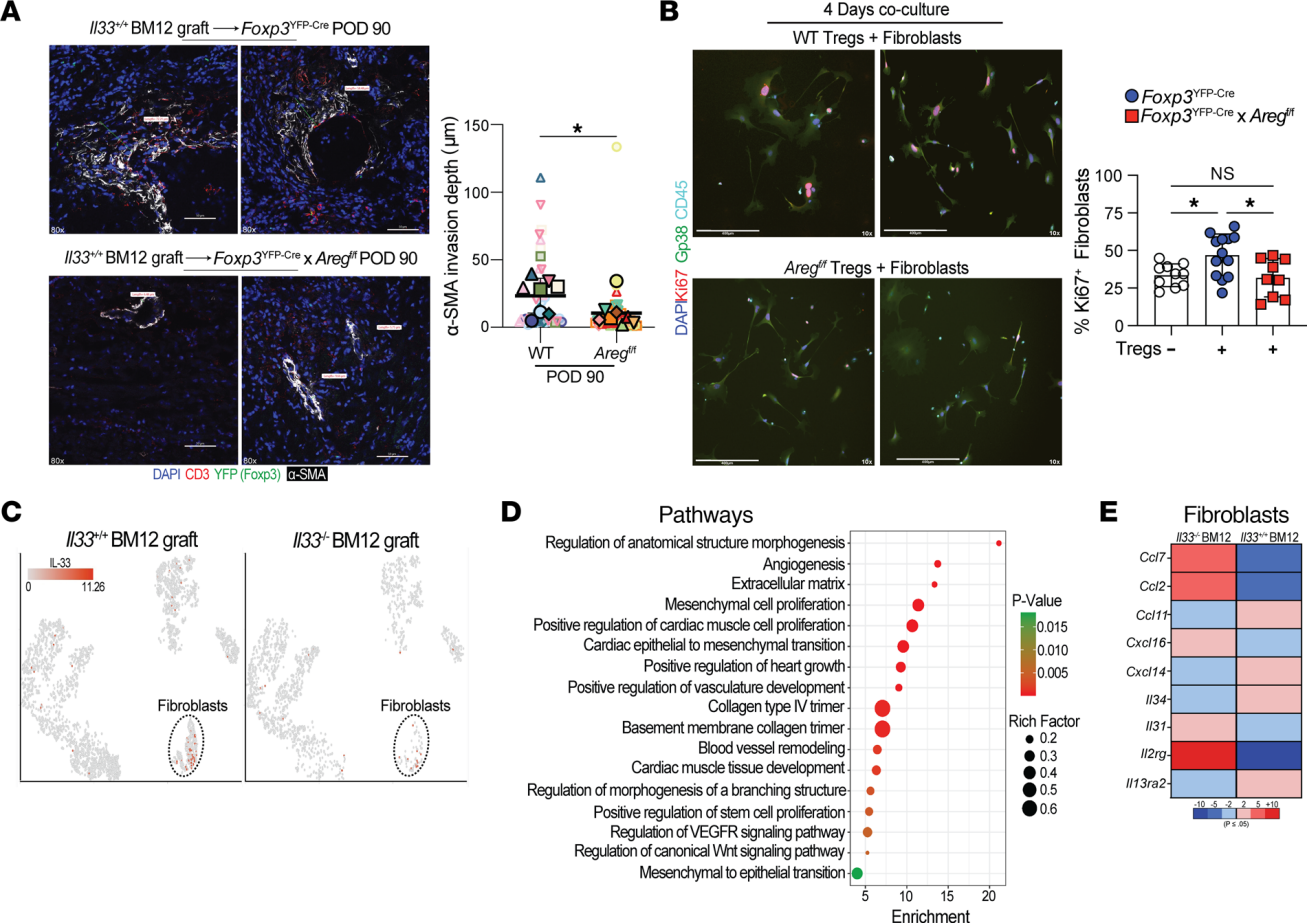
Treg-secreted Areg increases fibroblast proliferation. (**A**) Representative POD90 Bm12 heart grafts from *Foxp3*^YFP-Cre^ or *Foxp3*^YFP-Cre^ *Areg^fl/fl^* recipient mice stained for α-SMA (white), CD3 (red), Foxp3 (green), and DAPI (blue). Graph shows quantitative analysis of α-SMA invasion around blood vessels (7–9/group were evaluated, and 2 depths/graft were quantitated). The results represent cumulative from 4 independent experiments. Large symbols represent the mean counts from individual grafts, and color- and symbol-matched small symbols provide the values from each sample section. The thick bar and error bars represent the mean ± SD calculated from the biological sample means. **P ≤ 0.01, by 1-tailed Student’s *t* test. (**B**) Representative immunofluorescence images depicting Gp38 (green), Ki67 (red), CD45 (teal), and DAPI (dark blue) staining of B6 *St2^–/–^* fibroblasts following 4 days of coculturing with *Foxp3*^YFP-Cre^ control or *Foxp3*^YFP-Cre^ *Areg^fl/fl^* Tregs in the presence of IL-2 and IL-33. Data were pooled from 2 independent experiments (9–11/group), and each point is an individual well represented as the mean ± SD. *P ≤ 0.05, by Kruskal-Wallis test. (**C**) *t*-SNE projection of IL-33–expressing cells in *Il33^+/+^* or *Il33^–/–^* Bm12 grafts at POD14 (*n* = 3 hearts/group). (**D**) Gene set enrichment analysis of fibroblasts from *Il33^+/+^* or *Il33^–/–^* Bm12 grafts. (**E**) Heatmap depicts selected fibroblast-expressed molecules modulated by the presence of IL-33.

α–Smooth muscle actin (α-SMA) is a key indicator of activated cardiac fibroblasts that differentiate into myofibroblasts. This transformation, along with epithelial-mesenchymal transition, often occurs after heart injuries and contributes to fibrosis and heart failure through excessive production of fibronectin and collagen (37, 38). Heart grafts from *Foxp3*^YFP-Cre^ or *Foxp3*^YFP-Cre^ *Areg^fl/fl^* recipient mice were examined for increased α-SMA at POD50 and POD90 (Figure 7A). Areg expression by recipient Tregs increased α-SMA expressing fibroblasts around graft vasculature and promoted their invasion into the myocardium at the later POD90 time point (Figure 7A). To determine whether Treg-secreted Areg could directly potentiate fibrosis, Tregs from *Foxp3*^YFP-Cre^ or *Foxp3*^YFP-Cre^ *Areg^fl/fl^* mice were isolated and cocultured with primary fibroblasts from ST2*^–/–^* B6 mice with addition of IL-2 and IL-33. On day 4 of culture, the addition of Areg-competent Tregs increased fibroblast proliferation, as measured by IF staining for Ki67 (Figure 7B). This function of Treg was reduced by the deletion of Areg (Figure 7B). We observed similar reductions in Ki67 expression using flow cytometric assessment of fibroblasts from *Foxp3*^YFP-Cre^ *Areg^fl/fl^* Treg cocultures compared with fibroblasts from *Foxp3*^YFP-Cre^ (Supplemental Figure 8). Increased proliferation of fibroblasts by IL-33–stimulated Tregs coincided in vivo with more abundant fibroblasts in *Il33^+/+^* grafts compared with *Il33^–/–^* grafts (Figure 7C). Examination of the transcriptome of fibroblasts isolated from *Il33^+/+^* versus *Il33^–/–^* Bm12 grafts revealed enrichment for gene sets implicated in the proliferation of mesenchymal and cardiac muscle cells, collagen synthesis, Wnt, and vascular growth factor signaling (Figure 7D). Accordant with this was an IL-33–mediated increase in fibroblast expression of *Cxcl14* (Figure 7E), which recruits Treg (39), but also acts as a fibroblast autocrine growth factor (40). In total, our data suggest that IL-33–stimulated Areg production from Tregs promotes tissue remodeling after transplantation by stimulating fibroblast proliferation.

### Treg Areg secretion is negatively regulated by TCR stimulation and can be blocked with mTOR inhibition.

**Figure 8:**
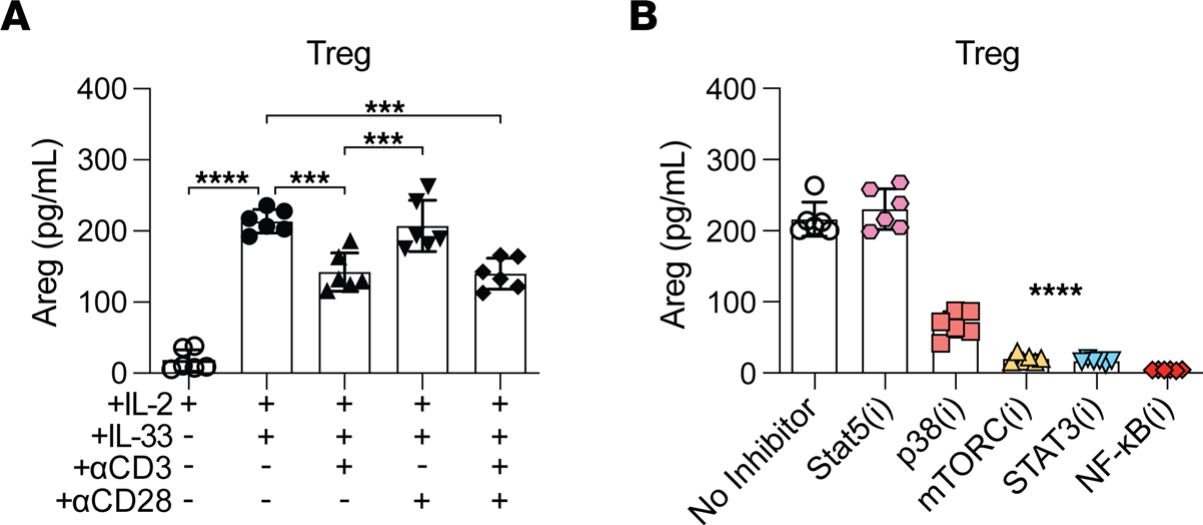
Modulation of Treg Areg secretion through ST2 and the TCR. (**A** and **B**) Tregs from *Foxp3*^YFP-Cre^ mice were cultured under various conditions: (**A**) different combinations of IL-33, plate-bound anti-CD3, and anti-CD28 antibody or (**B**) IL-33 and specific signaling pathway inhibitors (i). After 4 days of culturing, the Areg concentration was quantified by ELISA. The results represent cumulative data from 3 independent experiments, with a total of *n* = 6/group. Individual data points are depicted in the graphs, along with the group mean ± SD. ***P ≤ 0.005 and ****P ≤ 0.0001, by 1-way ANOVA with Tukey’s multiple-comparison test.

Treg function and persistence are shaped by environmental inputs like the availability of MHCII, costimulation, and local cytokines (41). The above data indicate that graft IL-33 played a significant role in initiating and sustaining Treg reparative functions. These functions are likely beneficial early on but need to be limited in order to prevent CR-causing fibrosis. We sought to define targetable pathways controlling Areg secretion. Treg production of Areg, while driven by IL-33, is independent of TCR stimulation (12). Consistent with these past observations, we observed the highest levels of Areg secretion by sorted Tregs with IL-33 alone (Figure 8A). While CD28 costimulation of Tregs did not modulate Areg levels, TCR-stimulation significantly decreased it compared with IL-33 stimulation alone (Figure 8A). IL-33 activation of p38 and NF-κB activation downstream of ST2 support Treg proliferation (42). Other pathways implicated in ST2-driven immune cell effector molecule secretion include mTOR, NF-κB, and STAT3 (43, 44). When these signaling pathways were targeted with inhibitors, IL-33–mediated Areg secretion was potently reduced (Figure 8B). This was in contrast to IL-2–activated STAT5, whose inhibition did not alter Areg secretion (Figure 8B). In total, these data establish that IL-33–stimulated Areg expression by Tregs was blunted by potent TCR stimulation and a clinically utilized immunosuppressant targeting mTOR.

## Discussion

Although proinflammatory alloreactive T cells and B cells have been identified as contributors to fibrosis and CAV, targeting these immune cells with immunosuppressants at levels leading to infections and malignancy in recipients has not solved the problem of CR (32, 45). We investigated other potential mechanisms that perpetuate CR, particularly failed or dysregulated tissue repair. It has been well documented that injured tissues release self-molecules containing DAMPs to initiate inflammatory responses (2, 3). It is clear, however, from our current studies and past findings (19) that IL-33 is unique among DAMPs, as it triggers regulatory and reparative pathways in both macrophages and Tregs. It is also evident in the current studies that, although IL-33 is protective immediately after transplantation (19), over time, IL-33–stimulated repair became dysregulated, causing fibrotic pathology in allografts. Tregs are generally viewed as a beneficial immune cell after transplantation (46), and our observations are compatible with prior studies showing that Treg production of Areg is critical for injury resolution (47). Given these contexts, the current findings establish an unexpected detrimental role for Tregs after transplantation and identify a CR-promoting mechanism that could potentially be therapeutically targeted. Our findings also have broad implications for the development of fibrosis in the presence of T cell responses to persistent antigens, such as in autoimmune disorders, chronic graft versus host disease, or systemic sclerosis.

In the MHCII-mismatched heart transplant allograft examined, populations of Tn, Teff, Trm, NK, and NKT cells were present, but their responses did not seem adequate to create an environment dominated by proinflammatory macrophages. Instead, macrophages expressing genes associated with alternative activation, phagocytosis, and tissue repair (21, 22, 48) were predominantly present. Genes used to define reparative macrophages, such as *Arg1*, *Mrc1*, *Mgl2*, and *Lyve1* were regulated by graft IL-33, thus IL-33 acted as a potent reparative DAMP that directed local macrophage differentiation.

Our scRNA-Seq analysis matches other reports that fibroblasts are a major source of IL-33 in cardiac tissues (49). Similarly, FRCs in the T cell zone of SLOs are IL-33^+^ and upregulate IL-33 further during inflammation to support CD8^+^ and CD4^+^ Th1 cell responses (29, 50). FRCs also create fibrotic niches in the SLOs, where T cells are activated and then leave the lymphoid tissues on FRC fibrotic networks (51–53). The study of SLOs has established the importance of fibrotic niches to generate immune responses, yet how immunological niches contribute to peripheral immune responses is less clear. IL-33 deletion from the lung adventitial vascular niches results in inadequate responses to helminths (54). Decreasing IL-33 in the aged muscle causes inadequate Treg-regenerative responses (13). Tregs localize with Teffs during inflammation via shared CXCR3 expression (55). While Tregs suppress excessive local inflammation (41), they also aid Trm generation by making TGF-β bioavailable to upregulate CD103 (56). Treg activation of TGF-β after epidermal injury also supports neutrophil responses by directing keratinocyte secretion of CXCL5 (57). Our findings reveal how IL-33 directs Tregs to drive local fibroblast proliferation via Areg but also supports fibroblast expression of *Cxcl14*, which may further recruit Tregs (39), and acts as a fibroblast autocrine growth factor (40). Overall, our data suggest a mechanism whereby Tregs support immune effector responses by generating fibrotic niches that provide a structure for immune cell infiltration and localization. Disrupting IL-33 signaling or targeting IL-33^+^ fibroblasts in perivascular niches may prevent allograft fibrosis and vasculopathy.

Tregs in nonlymphoid organs upregulate specialized gene programs in response to tissue-specific stimuli (25, 28). IL-33 is suggested to be important for tissue Treg generation, maintenance, and function. Tregs in visceral adipose tissue are reliant on the IL-33/ST2 axis to maintain their overall numbers (24), and IL-33 supports Treg-reparative functions after lung or skeletal muscle injury (13, 14). Yet, these prior studies used ST2- or IL-33–deficient mice, in which defects in Treg ontogeny versus local programming cannot be untangled. Using a model in which IL-33–deficient grafts are placed in WT recipients, we definitively established that, upon tissue infiltration, IL-33 stimulated transcripts associated with residency, survival, activation, and repair in Tregs. Tissue Treg accumulation depends on TCR recognition of peptide-MHCII (58). For Bm12 allografts in B6 recipients, the lone AlloAg is I-A^Bm1^, which is found on donor antigen-presenting cells (APCs) and graft endothelial cells (59, 60). Infiltrating Tregs may also recognize self-peptides on the closely related I-A^bm12+^ or I-A^b^ on recipient-derived local APCs. *Nr4a1* (Nur77) is used to quantitate how strongly the TCR of T cells is stimulated (61). We saw IL-33 increase *Nur77* expression when Tregs migrated to allogeneic, but not syngeneic, cardiac tissues (Figure 3I). This too suggests that IL-33 functions with Treg recognition of I-A^bm12+^ and parallels our recent findings that IL-33 intensifies weak alloreactive TCR signaling in CD4^+^ T cells (29). Thus, IL-33 is an important local stimulus that can boost TCR signaling in tissues where APCs or cognate peptide-MHCII may be limited. Our in vitro data demonstrate that potent anti-CD3 stimulation on Tregs negatively regulated Areg secretion (Figure 6A). Thus, as antigen concentrations decrease, IL-33 signals will support Treg-reparative functions. This premise is further supported by the observation that Areg levels were highest in syngeneic grafts (Figure 3H). Such a mechanism may be particularly relevant in transplantation, as the recipient’s immune system eliminates donor-derived APCs to reduce TCR stimulation, thereby promoting subsequent IL-33–induced Treg repair activities.

Efforts to harness suppressive Tregs as cell therapies clinically are underway (46, 62). Their capacity to lessen immunosuppression or induce tolerance after solid organ transplantation is clear from preclinical studies, however, potential unexpected side effects due to dysregulated tissue repair functions need to be considered. Elevated Areg levels are linked to pathogenic airway remodeling and fibrotic lung allograft dysfunction (16), and Areg is increased in epithelial cells of patients with asthma and during chronic obstructive pulmonary disease, where it promotes epithelial cell proliferation and mucin production (63–65). While Areg expression is typically absent in healthy livers, it becomes elevated during acute damage and in cases of liver cirrhosis (66). Thus, the potential risk of dysregulated Treg cell therapy–mediated, Areg-driven repair after heart, lung, and liver transplantation may exist. Incorporated tags and kill switches in clinical Treg products allow their elimination in the case of overimmunosuppression or Teff contamination or conversion. These tools may be called upon to prevent pathologic repair if necessary.

Bm12 survival is dependent on immune regulation by Tregs, as demonstrated by Schenk et al., who showed that targeting Tregs via anti-CD25 antibodies resulted in rejection of Bm12 grafts (17). Areg is implicated in the suppressive functions of Tregs (33), with Areg derived from mast cells mediating EGFR-dependent stimulation of Tregs that is needed for regulation of local immune responses (34). We found no differences in systemic CD4^+^ or CD8^+^ T cell activation, memory, or differentiation in *Foxp3*^YFP-Cre^ *Areg^fl/fl^* Treg recipients of BM12 allografts when compared with *Foxp3*^YFP-Cre^ controls (Supplemental Figure 5). Nor did we find a defect in the ex vivo suppressive function of *Foxp3*^YFP-Cre^ *Areg^fl/fl^* Tregs (Supplemental Figure 4C). In addition to Tregs (12), numerous immune cells express Areg in the context of inflammatory stimuli, including tumor-infiltrating CD8^+^ T cells (67), Th2 cells, macrophages, and type 2 innate lymphoid cells (ILC2s) (15). In our current studies, we found that over time, Tregs contributed to local pathology leading to CR through Areg secretion. This protective effect in the absence of Treg-secreted Areg argues against the importance of autocrine Areg for Treg-suppressive functions in the graft, as it would be expected that a lack of sufficient Treg-suppressive capacity would result in Bm12 allograft rejection (17). Moreover, our current data are consistent with unaltered immune responses to infections in *Foxp3*^YFP-Cre^ *Areg^fl/fl^* mice (12). While Tregs are a dominant source of Areg, other immune cells may act as a sufficient source of Areg for Treg-suppressive function. Clearly, Areg is an important effector molecule utilized by Tregs to shape outcomes after HTx, but the development of more precise models, such as those allowing inducible deletion of Treg-expressed Areg versus EGFR will be required to define their specific effect on suppression of antigen-specific T cells and/or local allograft repair.

Innate and adaptive immune cells work together in an orchestrated manner to respond to injuries that often involve pathogen removal balanced with tissue repair. The continuous presence of a T cell response to local antigens, such as auto or AlloAgs, leads to pathology due to a persisting destructive proinflammatory response. The current study reveals how a dysregulated repair response involving interactions between recipient Tregs and IL-33^+^ fibroblasts in the allograft can contribute to the progression of CR. This is significant because Treg-mediated repair responses drove local fibrosis, which will decrease graft function, but also provided niches for the detrimental alloimmune response to further promote local pathology. The study highlights the importance of targeting this pathway after transplantation by utilizing existing inhibitor agents such as mTOR (i.e., sirolimus) or EGFR (i.e., dacomitinib) or agents that neutralize Areg after Treg-mediated repair is complete.

## Methods

### Sex as a biological variable.

Our mouse studies exclusively examined male donors and recipients to avoid immune responses to H-Y antigens that would be present in female recipients.

### Study design.

The study’s purpose was to elucidate whether local IL-33 directs immune-mediated tissue repair responses after transplantation. We hypothesized that recipient immune responses to MHC differences instigate persistent immune-mediated injuries that dysregulate repair over time. We applied scRNA-Seq in an MHC II–mismatched chronic rejection HTx model that used grafts deficient in the injury signal IL-33. This enabled us to understand the full scope of IL-33 in regulating immune remodeling in the tissues. IL-33 modulated over 700 genes in heart-infiltrating Tregs, including the growth factor Areg. When grafts in B6 *Foxp3*^YFP-Cre^ *Areg^fl/fl^* and *Foxp3*^YFP-Cre^ recipients were assessed, the deletion of Areg in Tregs protected against allograft fibrosis, vasculopathy, and perivascular fibrosis. Thus, Treg-mediated tissue repair processes became dysregulated after transplantation, as hypothesized. Analyzed data reflect in vivo assessments, bioinformatics computational tools, and in vitro assays. Age- and sex-matched mice were randomly assigned to experimental groups, and the studies were not blinded. Experimental replicates and statistical analysis are indicated in figure legends.

*Clinical specimens:* Pediatric heart transplant recipient samples were obtained from a prospective study funded by the National Institute on Allergy and Infectious Diseases (NIAID), titled *Alloantibodies in Pediatric Heart Transplantation* (NCT0100531) (68–71). This study enrolled recipient patients from 8 pediatric heart centers in the United States and Canada. Patients received thymoglobulin induction therapy with maintenance immunosuppression comprising tacrolimus and mycophenolate mofetil. Corticosteroids were only used for routine maintenance immunosuppression in sensitized individuals with a positive donor-specific cross-match. All individuals underwent serial surveillance allograft EMBs at 1–2, 4, and 8 weeks, then at months 3, 4, 6, 9, and 12 after HTx, and then 1–2 times per year until the first of either the 5-year post-transplantation study visit, study withdrawal, or end of the study follow-up. Patients also underwent EMB if rejection was suspected and to assess resolution following the rejection treatment. Coronary angiograms were performed 12 months after transplantation and annually thereafter and were analyzed by a single blinded pediatric cardiologist at the study core angiography laboratory of Washington University in St. Louis (Missouri, USA). EMBs from pediatric heart transplant recipients with moderate/severe (*n* = 9 patients), mild (*n* = 7 patients), and minimal (*n* = 4 patients) overall severity of inflammation after the first year were assessed (overall rejection grading is provided in Supplemental Table 1). FFPE sections were obtained from blocks of heart allograft tissue acquired from adult recipients with failed heart allografts at the time of retransplantation. Sample grading is provided in Supplemental Table 1.

### Animals.

B6 [B6.129(Cg) *Foxp3^tm4(YFP/icre)Ayr^*/J; *Foxp3*^YFP-Cre^ and B6(C)-*H2*-*Ab1^bm12^*/KhEgJ; Bm12] were purchased from The Jackson Laboratory. B6 *ST2^–/–^*, *Il33^–/–^*, and Bm12 *Il33^–/–^* were described previously (19). B6 *Foxp3*^YFP-Cre^ *Areg^fl/fl^* were generated by crossing *Areg^fl/fl^* mice (see Supplemental Methods for detailed information) with *Foxp3*^YFP-Cre^ mice. All mice were housed in a specific pathogen–free animal facility in the University of Pittsburgh Division of Laboratory Animal Resources.

### Vascularized HTx.

Hearts were transplanted into recipients through end-to-side anastomosis of the donor’s ascending aorta and pulmonary artery to the recipient’s abdominal aorta and inferior vena cava as described previously (19). See Supplemental Methods for detailed information.

### Histological, immunohistochemical, and immunofluorescence analyses.

See Supplemental Methods for information on processing and data analysis.

### Treg isolation and ex vivo assessments.

See Supplemental Methods for information on processing and data analysis.

### scRNA-Seq of transplanted hearts.

See Supplemental Methods for detailed information. In brief, mouse allografts were digested and homogenized using a Miltenyi tissue dissociator. Cells were further isolated using density gradients and Miltenyi Biotec Debris removal solution. Samples were uniquely hash tagged (77.5% ± 18.8% post-hashing viability), and a 10x Genomics chromium instrument was used to generate single-cell droplets to create scRNA libraries. Sequence data were processed using CellRanger and analyzed with Partek flow. Trajectory analysis was performed using Monocle 2, and cell-cycle scoring was performed using the Seurat R package.

### Statistics.

Significance determinations were completed using GraphPad Prism 9 (GraphPad Software). Statistical differences between groups were determined utilizing the tests indicated in each figure legend, with results considered statistically significant if *P* was 0.05 or less. For comparisons between 2 groups, a 2-tailed, unpaired Student’s *t* test or Mann-Whitney *U* test was used. For 3 or more groups, a 1-way ANOVA with Tukey’s multiple-comparison test or Kruskal-Wallis test was used. For studies involving biological replicates, we used Beeswarm SuperPlots (72), in which biological replicates were color and symbol coded. The large symbols depict a biological sample mean, and small versions of the color-matched symbol present the value for each individual biological replicate. A thick bar and error bars represent the mean ± SD calculated from the biological sample means. 

### Study approval.

All procedures completed at the University of Pittsburgh were approved by the IACUC of the University of Pittsburgh (protocol 22051178) and complied with the NIH’s *Guide for the Care and Use of Laboratory Animals* (National Academies Press, 2011). The CTOTC-04 study protocol was approved by the IRBs of all participating institutions and written informed consent was obtained from parents or guardians with child assent obtained according to local IRB policy and adult heart transplant samples were obtained and utilized with IRB approval from the University of Pittsburgh (STUDY19010210).

### Data and materials availability.

Raw scRNA-Seq data have been deposited in the Gene Expression Omnibus (GEO) database (GEO GSE235871). Supporting data values and all other data needed to evaluate the conclusions of this work are available in the manuscript, the supplemental materials, or the Supporting Data Values file.

## Author contributions

JJPW, LF, XZ, SW, TL, LRM, GKD, MAWT, ST, AL, KJB, HS, JBS, ELC, MAR, and TT performed experiments and analyzed data. LRM performed mouse breeding and genotyping. SW and TL completed surgical procedures. XZ, JBS, and ELC characterized and conducted quality control of the generated transgenic mice. LF, JJPW, KIA, and SMS completed bioinformatics analysis. RL, SAW, and SCW trained and supervised personnel and edited the manuscript. AJD provided pathologic assessments of clinical specimens. JJPW, LF, XZ, ELC, SMS, KIA, and HRT wrote the manuscript and generated figures and tables. HRT conceived the idea, designed the experiments, analyzed the data, trained and supervised personnel, and wrote and edited the manuscript. LF, JJPW, and XZ share the designation of co–first authors due to the important contributions to the studies and generation of the manuscript. JJPW is listed first among the co–first authors for his substantial efforts in the generation and revision of the manuscript, as well as his analysis of scRNA-Seq data, completion of the majority of presented experiments, as well as figure generation. LF is listed second among the co–first authors due to her generation of the first draft of the manuscript and completion of the scRNA-Seq studies that shaped the foundation of the study. XZ is listed third among the co–first authors for the generation and characterization of the mice that enabled the study and for the generation of important data using these tools.

## Supplementary Material

Supplemental data

Supplemental data set 1

Supplemental data set 2

Supplemental data set 3

Supplemental table 1

Supporting data values

## Version 1 12/02/2024

Electronic publication

## Version 2 12/20/2024

updated figure 7 and higher res figure 1
