# Supplementary material for: Dysregulated Treg repair responses lead to chronic rejection after heart transplantation
Source: J Clin Invest. 2024 Dec 2;134(23):e173593. doi: 10.1172/JCI173593 (PMC11601918; doi:10.1172/JCI173593)
Supplement: Supplemental data [file jci-134-173593-s009.pdf]

Supplementary Materials for

Dysregulated regulatory T cell repair responses lead to chronic rejection after heart transplantation

Jordan J. P. Warunek, Lu Fan, Xue Zhang ,Sihua Wang, Steven M. Sanders, Tengfang Li, Lisa R. Mathews, Gaelen K. Dwyer, Michelle A. Wood-Trageser, Stephanie Traczek, Andrew Lesniak, Kassandra Baron, Hailey Spencer Johnny Bou Saba, Emmanuel León Colón, Tracy Tabib, Robert Lafyatis, Mark A. Ross, Anthony J. Demetris, Simon C. Watkins, Steven A. Webber, Khodor I. Abou-Daya*, and Hēth R. Turnquist*

*Corresponding authors. Email: kha17@pitt.edu (KIA-D); het5@pitt.edu (HRT)

This PDF file includes:

Supplemental Materials and Methods

Legends and Figs. S1 to S8

Other Supplementary Material for this manuscript includes the following:

Data File S1-S3 (.xlsx)

Table S1 Overall Rejection Grading for Clinical Specimens (.xlsx)

SUPPLEMENTAL MATERIALS AND METHODS

Transgenic Mouse generation

Bm12 *il33*$^{-/-}$ mice were generated by backcrossing Bm12 mice, 6 times onto the *il33*$^{-/-}$ background at the University of Pittsburgh. Areg$^{tm1a(EUCOMM)Hmgu}$ (Tm1a) were obtained from the European Conditional Mouse Mutagenesis Program Consortium (Cat # ICS-HEPD0516_3_C04-1). Tm1a were crossed to flip recombinase transgenic mice from Jackson Laboratory (Cat # 005703; B6.Cg-Tg(ACTFLPe)9205Dym/J) to excise LacZ and Neo components. This resulted in germ-line-targeted *Areg*$^{fl/fl}$ mice (Tm1c). *Foxp3*$^{YFP-Cre}$x*Areg*$^{fl/fl}$ was generated by crossing *Areg*$^{fl/fl}$ (Tm1c) with *Foxp3*$^{YFP-Cre}$ (Cat # 016959; B6.129(Cg)-Foxp3tm4(YFP/icre)Ayr/J).

Vascularized heart transplantation

Bm12 donor hearts were transplanted into B6 *Foxp3*$^{YFP-Cre}$ or *FoxP3*$^{YFP-Cre}$ x *Areg*$^{fl/fl}$ as previously described (1, 2). For scRNA-seq: donor Bm12 *il33*$^{+/+}$ or Bm12 *il33*$^{-/-}$ hearts were transplanted into B6 recipients. Graft function was verified daily by abdominal palpation of heart contractions until the indicated day of harvest.

Histological and immunohistochemical staining

Tissues were fixed in 4% paraformaldehyde, paraffin-embedded, sectioned (5 µm) and stained with Masson's trichrome using standard protocols. Each specimen was cut into 2-3 sections at two different depths. Using QuPath, Open-source software for digital pathology image analysis, blue fibrosis areas were quantified using the train object classifier function as we have described (3). Trichrome percentage was quantified using

blue fibrosis areas (μm^2) divided by total area (μm^2). Vascular occlusion quantification was conducted with trichrome stained slides. SW and LF calculated percent occlusion by dividing the number of occluded vessels by the total number of vessels. For immunohistochemistry, sections were stained with either CD3 (Abcam ab11089), Foxp3 (Novus Biologicals NB100-39002), or CD11b (Abcam ab75476). Cell quantification was completed using QuPath's cell detection function to classify hematoxylin$^+$ cells and then the trainable object classifier to identify specific immune cells.

Quantitative immunofluorescence

Optimal cutting temperature compound-embedded (Fisher Scientific; 4585) frozen mouse heart transplants were sectioned at 5-μm, placed on glass slides, and stained for CD3 (Abcam; ab11089), GFP (Abcam; ab13970), CD11b (Abcam; ab75476), α-SMA (Sigma Aldrich; C6918), with fluorochrome-conjugated and host-specific secondary antibodies following established protocols from the Center for Biologic Imaging at the University of Pittsburgh (http://cbi.pitt.edu) as described previously (1). Images were analyzed in QuPath utilizing the cell detection function for DAPI$^+$ cells and then training the object classifier to detect immune cell staining for specific markers. For discriminating vessel versus myocardia immune cells, a 100 μm radius was drawn from each vessel adventitia and cells following inside or outside of these borders classified and quantitated.

Immunofluorescence and flow cytometry for Fibroblast Ki67

Primary fibroblasts isolated from the ears of B6 *St2*$^{-/-}$ mice were cultured and expanded for 10 days. Sort purified Tregs (CD4$^+$, CD127^{lo} CD25^{hi}, YFP-Foxp3$^+$) were obtained from

either B6 *Foxp3*$^{YFP-Cre}$ or *Foxp3*$^{YFP-Cre}$ x *Areg*$^{fl/fl}$ mice following conditioning with IL-33 (i.p. 0.5 μg/day for 7 days). Isolated Tregs (100,000 cells) were then cultured in 2-well chamber slides (Fisher Scientific 154461) with *St2*$^{-/-}$ fibroblasts (10,000 cells) for 4 days in complete RPMI media containing IL-2 (50 U/ml, Peptrotech #200-02) and IL-33 (100ng/ml, BioLegend #580508). Cells were fixed with 2% PFA, permeabilized with 0.1% Triton X-100, and stained with antibodies against Ki67 (Abcam ab15580), podoplanin (Invitrogen MA5-16113), CD45 (BD Pharmigen 550539). Secondary antibodies used were anti-Syrian hamster Cy2 (Invitrogen A21110), anti-rabbit Cy3 (Invitrogen A32794), and anti-rat Af647 (Jackson Immunoresearch 712-605-153). Cells were imaged using an EVOS FL Auto Tech (Life Technologies). Flow cytometry experiments were similarly completed after 3 days of culture using the antibodies BUV805 anti-mouse CD4 (BD OptiBuild #741912), BUV496 anti-mouse CD45 (BD OptiBuild #749889), PE-Cy7 anti-mouse Podoplanin (Biolegend #127412), PerCP-Cy5.5 anti-mouse Ki67 (Biolegend #652424).

Multiplex immunohistochemistry of clinical samples

Multiplex IHC for detecting Areg secreting Tregs (AREG/FOXP3/CD3/CD8) was conducted on selected FFPE slides. Briefly, after deparaffinization and antigen retrieval with ULTRA Cell Conditional Solution (CC1, pH 7.8, Roche, 950-224, Tucson, AZ) for 64 min at 100°C on the Ventana Discovery ULTRA. Slides were permeabilized with 0.01% Triton-X 100 for 20 min at RT followed by incubations with consecutive rounds of primary and paired secondary HRP-tagged secondary antibodies followed by fluorescently bound tyramides. Denaturation with ULTRA Cell Conditioning CC2 (pH 6, Roche, 950-223) was used between staining rounds on a Roche Ventana Discovery ULTRA to reduce cross-

reactivity. Nuclei were counterstained with DAPI (Thermo Scientific) at 1:1000 for 20 minutes and coverslipped with Gelvatol. Images were captured using Zeiss Axioscan Z.1 equipped with a 16-bit Hamamatsu CMOS monochrome camera for fluorescence acquisition. All antibody details are provided in the supplemental table.

Table. Antibodies and Incubations for Immunohistochemistry

Primary Antibody				Secondary Ab	Tertiary Detection		
**Target (clone)**	**Catalog Info (RRID*)**	**Dilution**	**Incubation**		**Reagent**	**Dilution**	**Incubation at RT**
AREG (polyclonal)	Millipore Sigma[1], HPA008720 (RRID: AB_1844978)	1:200	37°C, 16 min	DISC. OmniMap Anti-RB HRP (RUO; Roche[2], 760-4311, RRID: AB_2811043)	DISC. Rhodamine 6G Kit (RUO; Roche, 760-244)	predilute	32 min
FOXP3 (236A/E7)	Abcam[3], ab96048 (RRID: AB_10861686)	1:1000	RT, 2 hrs	Anti-Mouse HQ+Anti-HQ HRP	DISC. DCC Kit (RUO; Roche, 760-240)	predilute	32 min
CD3 (polyclonal)	Agilent/Dako[4], A045229-2 (RRID: AB_2335677)	1:200	37°C, 32 min	DISC. OmniMap Anti-RB HRP	DISC. Cy5 Kit (RUO; Roche, 760-238)	predilute	40 min
CD8 (C8/144B)	Agilent/Dako, M710301-2	1:1000	RT, 4 hrs	DISC. OmniMap Anti-MS HRP (RUO; Roche[4], 760-4310, RRID: AB_2885182)	DISC. FAM Kit (RUO; Roche, 760-243)	predilute	20 min

*RRID = Research Resource Identifier, [1]Millipore Sigma, St. Louis, MO; [2]Roche, Indianapolis, IN; [3]Abcam, Waltham, MA; [4]Agilent/Dako, Santa Clara, CA.

Splenocyte Treg isolation

Spleens were harvested from *Foxp3*$^{YFP\text{-}Cre}$ or *Foxp3*$^{YFP\text{-}Cre}$x*Areg*$^{fl/fl}$ mice and processed via mechanical dissociation through a 70-µm strainer and red blood cell lysis buffer to produce single-cell suspensions. $CD4^+$ T cells were purified from cell suspensions via negative

selection. Non-CD4$^+$ cells were labeled with anti-CD11b (Biolegend; 101202), anti-TER-119 (Biolegend; 116202), anti-Gr-1 (Biolegend; 108402), anti-I-A/I-E (Biolegend; 107602), anti-CD8α (Biolegend; 100702) and anti-B220 (Biolegend; 103202) mAbs. After incubation with Dynabeads™ Untouched™ Mouse CD4 Cells Kit (Invitrogen; 11416D), bead-bound cells were removed by magnetic isolation. Pure Tregs were next obtained by sorting based on CD4$^+$ CD127^{lo} CD25$^+$ and YFP-Foxp3 expression using a BD Aria II.

Suppression Assay

Fresh splenic Tregs (CD3$^+$ CD4$^+$ CD25$^+$ Foxp3$^+$) were sort purified from C57BL/6 *Foxp3*$^{YFP-Cre}$ or *Foxp3*$^{YFP-Cre}$ x Areg$^{fl/fl}$ mice. CD4$^+$ Tconvs (CD3$^+$ CD4$^+$ CD25$^-$ Foxp3$^-$) and CD8$^+$ CTLs (CD3$^+$ CD4$^-$ CD8$^+$) were also sorted from accompanying donors. CD4$^+$ and CD8$^+$ T cells were labeled with CellTrace Violet Cell Proliferation kit (Invitrogen; C34557), seeded at a concentration of 1x10^5/well in 96-well plates in complete RPMI and cultured for 4 days with or without Tregs in the presence of T cell activating CD3/CD28 Dynabeads (Gibco; 11456D) at a concentration of 1 bead:2 cells. Percent suppression of proliferating CD4$^+$ and CD8$^+$ T cells was quantified by flow cytometry and normalized to the minimum (no beads) and maximum (no Tregs) proliferation.

ELISA

Purified Tregs and CD4$^+$ conventional T cells (Tconvs) were plated in a round-bottom 96-well plate and cultured for 4 days in complete RPMI with 50 units/mL hIL-2 (Peprotech; 200-02-100ug) and 100 ng/mL recombinant mouse IL-33 (Biolegend; 58050). Areg levels in supernatant were quantified using an ELISA (R&D; DY989) according to the manufacturer's protocol.

Primary fibroblasts isolation and culture

Primary fibroblasts were isolated from B6 *St2*$^{-/-}$ mice ear tissue as described previously (4). Fibroblasts were cultured until confluent in RPMI 1640 medium supplemented with 10% fetal calf serum (FCS), 50 µM 2-mercaptoethanol, 100 µM asparagine, 2 mM glutamine and 1% penicillin-streptomycin solution.

In vitro assessment of the regulation of inhibition of Treg secretion of Areg

Tregs were purified and sorted from the spleens and lymph nodes of IL-33 pretreated *Foxp3*$^{YFP\text{-}Cre}$ mice (0.5 µg/d, i.p. for 10 days) and cultured in different combinations of plate-bound anti-CD3 (1 µg/mL), soluble CD28 antibody (5 µg/mL), hIL-2 (50 U/mL, Peprotech; 200-02) or recombinant mouse IL-33 (100 ng/mL, Biolegend; 58050), for 4 days and Areg in culture supernatant was quantified by ELISA. In related experiments, sorted Tregs were cultured in complete RPMI with 50 U/mL hIL-2, 100 ng/mL recombinant mouse IL-33 (Biolegend; 58050), and with or without the following inhibitors; *p38 inhibitor*: SB203580 (5 µM; Cell Signaling), *mTORC inhibitor*: rapamycin (25 nM; Millipore Sigma), *STAT3 inhibitor*: AG-490 (10 µM; Cell Signaling) and JSI-124 (200 nM; Tocris Bioscience), *STAT5 inhibitor*: CAS 285986-31-4 (1µM; Calbiochem) and *NF-κB inhibitor*: MG132 (5 µM; Sigma-Aldrich) and TCPA-1 (5 µM; Sigma-Aldrich). Changes in Areg secretion were assessed by ELISA (R&D; DY989).

Single cell RNA-seq of transplanted mouse hearts

Harvest: Mice were euthanized and immediately perfused with PBS containing 0.5% heparin via the left ventricle. Heart tissue was minced by scissors and then placed in 10ml RPMI digestion buffer (10% FBS, 12.5mg collagenase IV (Thermo Fisher; 17104019) and DNAse I (Sigma Aldrich; DN25-1G)) and further homogenized using Miltenyi GentleMacs C tubes and Miltenyi tissue dissociator. The tissue homogenate was incubated in a shaker at 250 rpm and 37°C for 45 minutes. After digestion, homogenate was passed through a 70 µm strainers, centrifuged, and immune cells were then separated by a density gradient using Lympholyte-M (Cedarlane; CL5031) according to the manufacturer's protocol. Interphase cells were isolated, washed with RPMI, and then further purified using Miltenyi Biotec Debris removal solution (130-109-398) per the manufacturer's instructions.

Multiplexing different samples: Isolated cells from each heart digest were resuspended in FACs buffer containing Fc block (101319, BioLegend) and incubated for 10 minutes at 4˚C. Each sample was next labeled with 0.5 µg of a unique Cell Hashing antibody (TotalSeq A0301-A0306 anti-mouse Hashtag 1-6, BioLegend), then incubated for 30 minutes at 4˚C. Cells were washed 3 times in 1 ml buffer PBS +2% FBS, pooled and washed in 1 ml PBS +2% FBS, before resuspension in 25ul of PBS +2% FBS. scRNA-seq was performed using a 10x Genomics Chromium instrument and 10X Genomics Single Cell 3' kit, version 3 according to the manufacturer's instructions with approximately 3000 cells loaded for each sample. mRNA and hashtag oligo cDNA libraries were generated and quantified (KAPA Library Quantification Kit, Illumina), characterized on a bioanalyzer, and sequenced using a Novaseq 6000 System (Illumina) sequencing (UPMC Genome Center).

Sequenced cells had a mean read count of about 60,000 reads per cell. The resultant sequencing data were preprocessed with Cell Ranger v4.0.0. The sequencing reads were aligned to mm10-2020-A (Mus musculus) reference genome. Cell ranger output files were uploaded into Partek Flow for further processing and analysis.

Single-cell RNA sequencing analysis and visualization

Files were imported into Partek flow version 10.0.23.0425, the dataset was then filtered to remove potential doublets and low-quality and dead cells using the following criteria: only cells with gene expression counts between 1,500 and 15,000, detected expressed genes between 400 and 4,000, and mitochondrial counts less than 20% were included in downstream analyses. The expression matrix was then normalized to the binary logarithm of counts per million (CPM) plus one, $\log_2(CPM+1)$. Dimensionality reduction was performed in two steps. First, a principal components analysis (PCA) was done under default conditions. The principal components that account for most of the variance in the dataset were determined using a scree plot of the eigenvalues vs ascending order of principal components. In this dataset, the scree plot's elbow point was at eight principal components. Second, to generate an adequately resolved projection of the dataset in two dimensions, a t-SNE plot was generated using the following settings (8 principal components, perplexity set to 40, 10,000 iterations, and all other parameters default). The resulting 2D t-SNE plot was then used to annotate the projected neighborhoods by combining graph-based clustering (**fig. S1D**) (12 principal components, 1,000 iterations per random start, and all other parameters default) and feature gene expression (**Fig. 1B-C,**

fig. S1). Enrichment of active genes from previously published gene sets (5) in each annotated cell type was determined using AUCell (**Fig. 3C**).

Differential gene expression analysis (DEG) between *il33*$^{+/+}$ and *il33*$^{-/-}$ samples within each annotated cell type was performed using Partek's GSA ("gene specific") algorithm with default settings. For each cell type, a list of differentially expressed genes (|fold change| >=2 and p-value<=0.05; **Figs. 2A, 3A**) were analyzed with a gene set enrichment analysis (GSEA) (Figs 3B, 7D). Trajectory analysis was performed using Monocle 2 under default settings, except with "scaling = FALSE". Trajectory analysis was limited to cells annotated as monocytes or macrophages (**Fig. 2B-C**). Multiple DEGs were then performed using GSA to identify differentially expressed genes of each cell state uncovered by the trajectory analysis (**Fig. 2D-E**).

Single-nuclei RNA sequencing analysis and visualization

Dataset GSE203548 was downloaded from GEO then imported to Partek Flow and processed similarly to our generated dataset except after normalization the samples were integrated using Seurat3 integration to minimize batch effect. Following processing and projection, the nuclei neighborhood that expressed PTPRC (CD45), CD5, CD3E/G/&D (CD3) was selected for re-projection using UMAP. Differential gene expression of CD4, CD8, FOXP3, IKZF2, CCR7, CD44, SELL (CD62L), CD69, and KLRG1 was assessed visually on the UMAP to classify the neighborhoods of T cells.

Flow Cytometry

<u>Splenocyte Assessments:</u> Isolated splenocytes were blocked with cell staining buffer (PBS containing 5% fetal bovine serum (FBS)) with 5% normal goat serum (NGS) and then stained with the following antibodies: anti-mouse CD3e (clone 145-2C11, BD Pharmingen; clone 17A2, Biolegend), anti-mouse CD4 (clone RM4-5, BD Horizon), anti-mouse CD8 (clone 53-6.7, BD Pharmingen), anti-mouse CD25 (clone PC61, BD Horizon), anti-mouse CD44 (clone IM7, BD Horizon), anti-mouse CD45 (clone 30-F11, Biolegend), anti-mouse CD62L (clone MEL-14, BD Pharmingen), anti-mouse CD69 (clone H1.2-F3, BD Pharmingen), anti-mouse ST2 (clone U29-93, BD Pharmingen). For intracellular staining, Intracellular Fixation & Permeabilization buffer set (eBioscience) were used overnight to fix and permeabilize the cells. Surface-stained cells were then stained with the following antibodies in perm buffer (PBS containing 1% FBS, 1g/L NaN3 and 1g/L Saponin) containing 5% NGS: anti-mouse GATA3 (clone L50-823, BD Pharmingen), anti-mouse Foxp3 (FJK-16s, Invitrogen), anti-mouse T-bet (clone 4B10, Biolegend), anti-mouse RORγt (clone Q31-378, BD Horizon), anti-mouse Ki-67(clone 16A8, Biolegend). All samples were acquired with a Fortessa or LSRII (BD Biosciences) and data were analyzed with FlowJo (BD Biosciences).

<u>Assessment of graft-localized immune cells:</u> 3 μg of fluorochrome-conjugated CD45 antibodies were injected i.v. into mice 3 minutes before tissue harvest. Heart grafts were excised, cut into fragments, and homogenized using a gentleMACS Dissociator (Miltenyi Biotec) in digestion media (RPMI, 2% FBS) containing 350 U/mL type IV collagenase (Gibco) and 20 μg/mL DNase I (MilliporeSigma). Digests were then passed through 70

µm cell strainers and the resulting single-cell suspensions were purified with a percoll gradient (37.5% Percoll Cytiva) to obtain mono-nuclear cells. Spleens were isolated and single-cell suspensions generated following mechanical dissociation and RBC lysis. Cells were stained in PBS containing extracellular antibodies and eBioscience Fixable Viability Dye. After washing, cells were fixed with 2% PFA for 10 minutes at room temperature. For intracellular staining, cells were permeabilized with 0.5% Saponin. Data was acquired using an Aurora (Cytek Biosciences) and analyzed using FlowJo v10.9.0 (BD Life Sciences). Flow antibodies**:** SB436 anti-mouse CD3e (1:100 Invitrogen Cat: 62-0031-82; Clone: 145-2C11), BUV563 anti-mouse CD4 (1:200 BD Horizon Cat: 612923; Clone: GK1.5, Pecy5 anti-mouse B220 (1:200 BD Pharmingen Cat: 553091; Clone: RA3-6B2), APC-eFluor780 anti-mouse CD11b (1:200 Invitrogen Cat: 47-0112-82; Clone: M1/70), BV510 anti-mouse CD11c (1:200 BioLegend Cat: 117338; Clone: N418), BUV395 Rat anti-mouse MHCII (IA/IE) (1:200 BD OptiBuild Cat: 743876; Clone: 2G9), BUV661 anti-mouse F4/80 (1:200 Invitrogen Cat: 376-4801-80; Clone: BM8), BV785 anti-mouse Ly-6C (1:200 BioLegend Cat: 128041; Clone: HK1.4), BUV805 anti-mouse Ly-6G (1:200 BD OptiBuild Cat: 741994; Clone: 1A8), BUV737 anti-mouse IL33R (ST2) (1:100 BD OptiBuild Cat: 749323; Clone: U29-93), Pecy7 anti-mouse LYVE1 (1:100 Invitrogen Cat: 25-0443-80; Clone: ALY7), PE anti-mouse Folr2 (1:100 BioLegend Cat: 153303; Clone: 10/FR2), BV421 anti-mouse CD45.1 (1:200 Invitrogen Cat: 404-0453-80; Clone: A20), BUV496 anti-mouse CD45.2 (1:200 Invitrogen Cat: 364-0454-80; Clone: 104), PE-Cy5.5 anti-mouse CD8a (1:200 Invitrogen Cat: 35-0081-80; Clone: 53-6.7), Pacific Orange anti-mouse CD45 (I.V. 3ug Invitrogen Cat: MCD4530; Clone: 30-F11), FITC anti-mouse Foxp3 (1:100 BD Pharmingen Cat: 560403; Clone: MF23), PE-Dazzle 594 anti-mouse CD301b

(1:300 BioLegend Cat: 146816; Clone: URA-1), Biotin anti-mouse Areg (1:100 R&D Systems Cat: BAF989; Polyclonal), PercpCy5.5 Streptavidin (1:100 BioLegend Cat: 405214), eFluor450 anti-mouse Nur77 (1:200 Invitrogen Cat: 48-5965-82; Clone: 12.14), AF647 anti-mouse/human TCF1/7 (1:100 Cell Signaling Technology Cat: 6709S; Clone: C63D9), eBioscience Fixable Viability Dye eFluor 506 (1:500 Thermo Fisher Cat: 65-0866-14).

Supplemental References:

1. Li T, Zhang Z, Bartolacci JG, Dwyer GK, Liu Q, Mathews LR, et al. Graft IL-33 regulates infiltrating macrophages to protect against chronic rejection. *J Clin Invest.* 2020;130(10):5397-412.

2. Turnquist HR, Zhao Z, Rosborough BR, Liu Q, Castellaneta A, Isse K, et al. IL-33 expands suppressive CD11b+ Gr-1(int) and regulatory T cells, including ST2L+ Foxp3+ cells, and mediates regulatory T cell-dependent promotion of cardiac allograft survival. *J Immunol.* 2011;187(9):4598-610.

3. Bartolacci JG, Behun MN, Warunek JP, Li T, Sahu A, Dwyer GK, et al. Matrix-bound nanovesicle-associated IL-33 supports functional recovery after skeletal muscle injury by initiating a pro-regenerative macrophage phenotypic transition. *NPJ Regen Med.* 2024;9(1):7.

4. Khan M, and Gasser S. Generating Primary Fibroblast Cultures from Mouse Ear and Tail Tissues. *J Vis Exp.* 2016;107(107):53565.

5. Munoz-Rojas AR, and Mathis D. Tissue regulatory T cells: regulatory chameleons. *Nat Rev Immunol.* 2021;21(9):597-611.

SUPPLEMENTAL FIGURES (S1-S8)

A

Cell Type	IL-33$^{+/+}$ BM12 Donor Hearts			IL-33$^{-/-}$ BM12 Donor Hearts			Counts			Percentages			
	IL-33 WT #1	IL-33 WT #2	IL-33 WT #3	IL-33 KO #1	IL-33 KO #2	IL-33 KO #3	IL33 WT Total	IL33 KO Total	Combined	IL33 WT Total	IL33 KO Total	Combined	Difference
B cells	45	26	105	91	4	15	176	110	286	6.40%	6.60%	6.40%	-0.20%
Tcm	42	19	53	62	49	41	114	152	266	4.10%	9.10%	6.00%	-4.90%
cDCs	38	6	44	48	9	26	88	83	171	3.20%	4.90%	3.90%	-1.80%
Teff/em	54	23	86	64	42	58	163	164	327	5.90%	9.80%	7.40%	-3.90%
Fibroblasts	34	6	270	31	4	30	310	65	375	11.20%	3.90%	8.40%	7.40%
Unconv. T cells	25	2	27	18	7	4	54	29	83	2.00%	1.70%	1.90%	0.20%
Mo-DCs	37	4	111	28	7	17	152	52	204	5.50%	3.10%	4.60%	2.40%
Monocytes	82	5	15	33	7	12	102	52	154	3.70%	3.10%	3.50%	0.60%
Macrophages	192	10	98	71	21	37	300	129	429	10.90%	7.70%	9.70%	3.20%
Tn	19	14	78	102	15	15	111	132	243	4.00%	7.90%	5.50%	-3.80%
NK cells	140	53	475	105	33	125	668	263	931	24.20%	15.70%	21.00%	8.50%
NKT cells	50	17	100	69	28	83	167	180	347	6.00%	10.70%	7.80%	-4.70%
Trm-like	81	20	106	59	33	63	207	155	362	7.50%	9.20%	8.20%	-1.70%
Tregs	27	25	98	55	25	33	150	113	263	5.40%	6.70%	5.90%	-1.30%
**Total cells**	866	230	1666	836	284	559	2762	1679	4441				

B

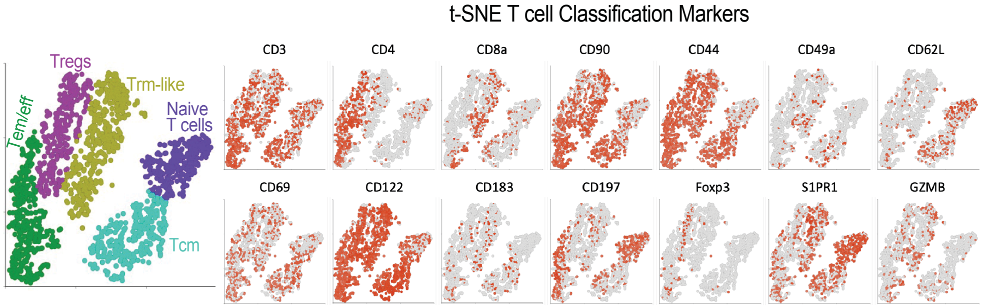

C

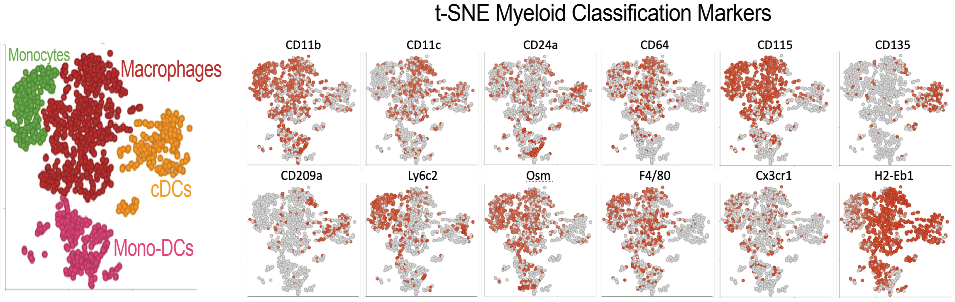

D

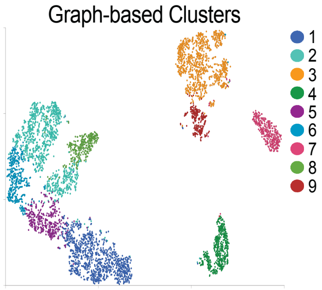

S1.**ScRNA-seq classification and quantification of immune subsets in transplanted hearts.** (**A**) Total cell counts from scRNA-seq comparing *il33*$^{+/+}$ and *il33*$^{-/-}$ Bm12 donor

hearts into wildtype C57BL/6 (B6) recipients 14 days after transplant (post-operative day (POD14); n=3/group). (**B-C**) t-SNE of T cell and myeloid immune populations and visualization of gene expression of the markers used for classification. (**D**) Graph-based projection with clusters.

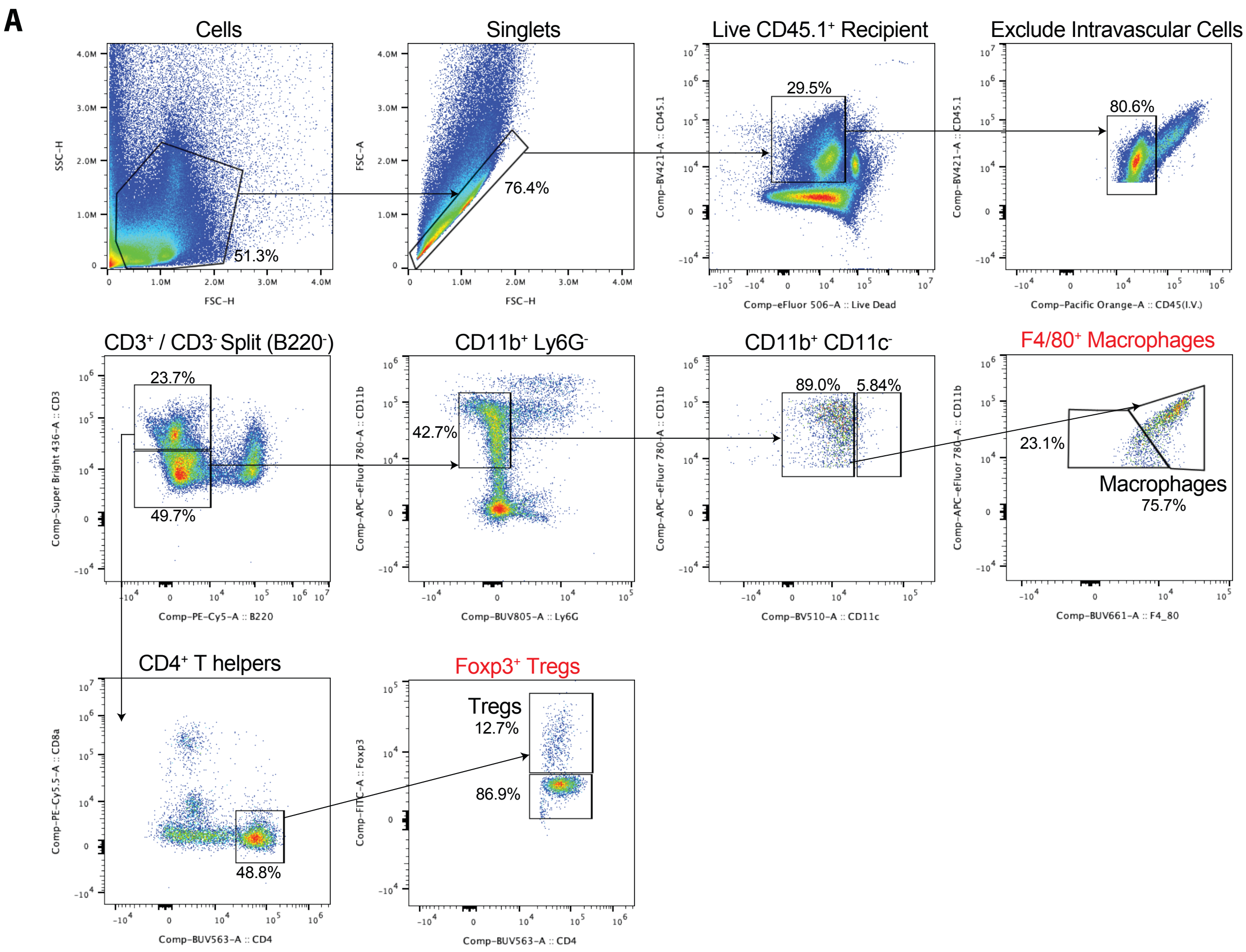

S2. **Flow Cytometry Gating Scheme.** Leukocytes were isolated from heart grafts and stained with antibodies to analyze Treg and macrophage cell populations.

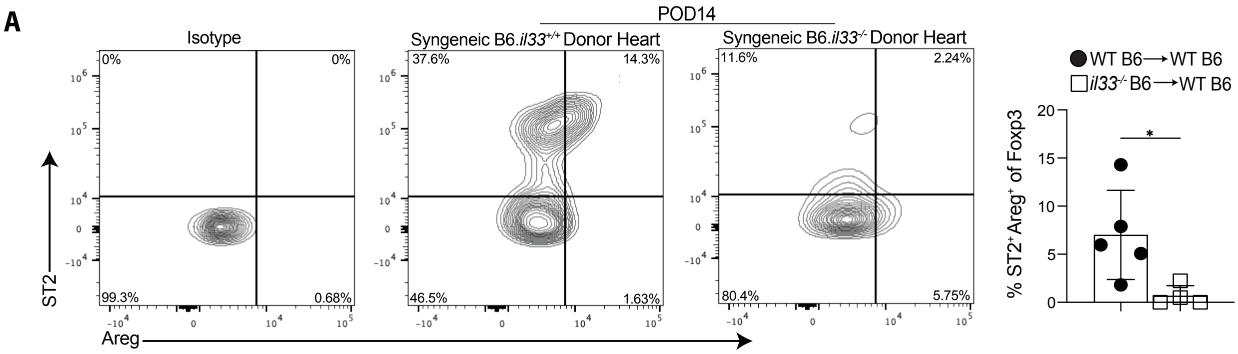

S3. **IL-33 in syngeneic heart grafts increases ST2$^+$ Areg$^+$ Tregs.** Syngeneic B6 *il33*$^{+/+}$ or *il33*$^{-/-}$grafts were transplanted into CD45.1 B6 mice and recipient Tregs were analyzed at POD14. n=4-5 grafts/group. Mean±SD. Student's *t* test *p≤0.05.

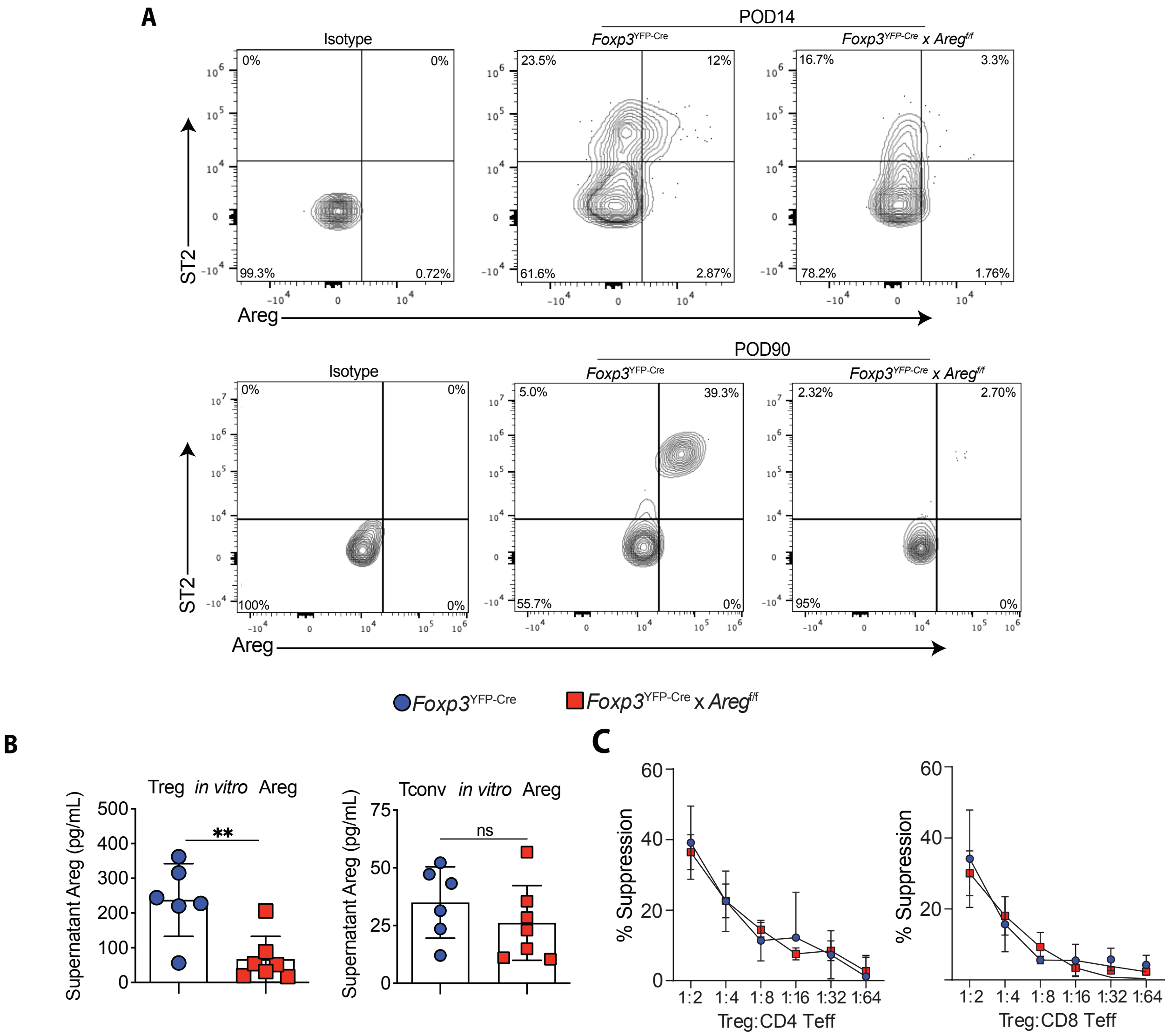

S4. **Deletion of Areg in *Foxp3*$^{\text{YFP-Cre}}$ x *Areg*$^{\text{fl/fl}}$ mice.** (**A**) Flow plots depicting the frequency of recipient ST2^{+} Areg^{+} Tregs following Bm12 transplant into B6 *Foxp3*$^{\text{YFP-Cre}}$ or *Foxp3*$^{\text{YFP-Cre}}$x*Areg*$^{\text{fl/fl}}$ recipients at POD14 and POD90. These data are representative of that observed in three separate experiments (**B**) Sort purified Tregs (CD3^{+} CD4^{+} CD25^{+} Foxp3^{+}) and CD4^{+} Tconv (CD3^{+} CD4^{+} CD25^{-} Foxp3^{-}) were isolated from B6 *Foxp3*$^{\text{YFP-Cre}}$ or *Foxp3*$^{\text{YFP-Cre}}$ x Areg$^{\text{fl/fl}}$ mice pre-conditioned with IL-33 (ip 1 μg/d) for 10 days. Tregs and Tconvs were cultured with IL-2 (50U/mL) and IL-33 (100 ng/mL) for 4 days and

supernatant Areg was quantified by ELISA. (**C**) Suppression assay with Tregs sort purified from B6 *Foxp3*$^{YFP-Cre}$ or *Foxp3*$^{YFP-Cre}$ x *Areg*$^{fl/fl}$ mice, alongside $CD4^+$ Tconv and $CD8^+$ cytotoxic T cells ($CD3^+$ $CD4^-$ $CD8^+$). Data are pooled from two independent experiments with a total of n=6 wells/group. Student's *t* test **p≤0.01.

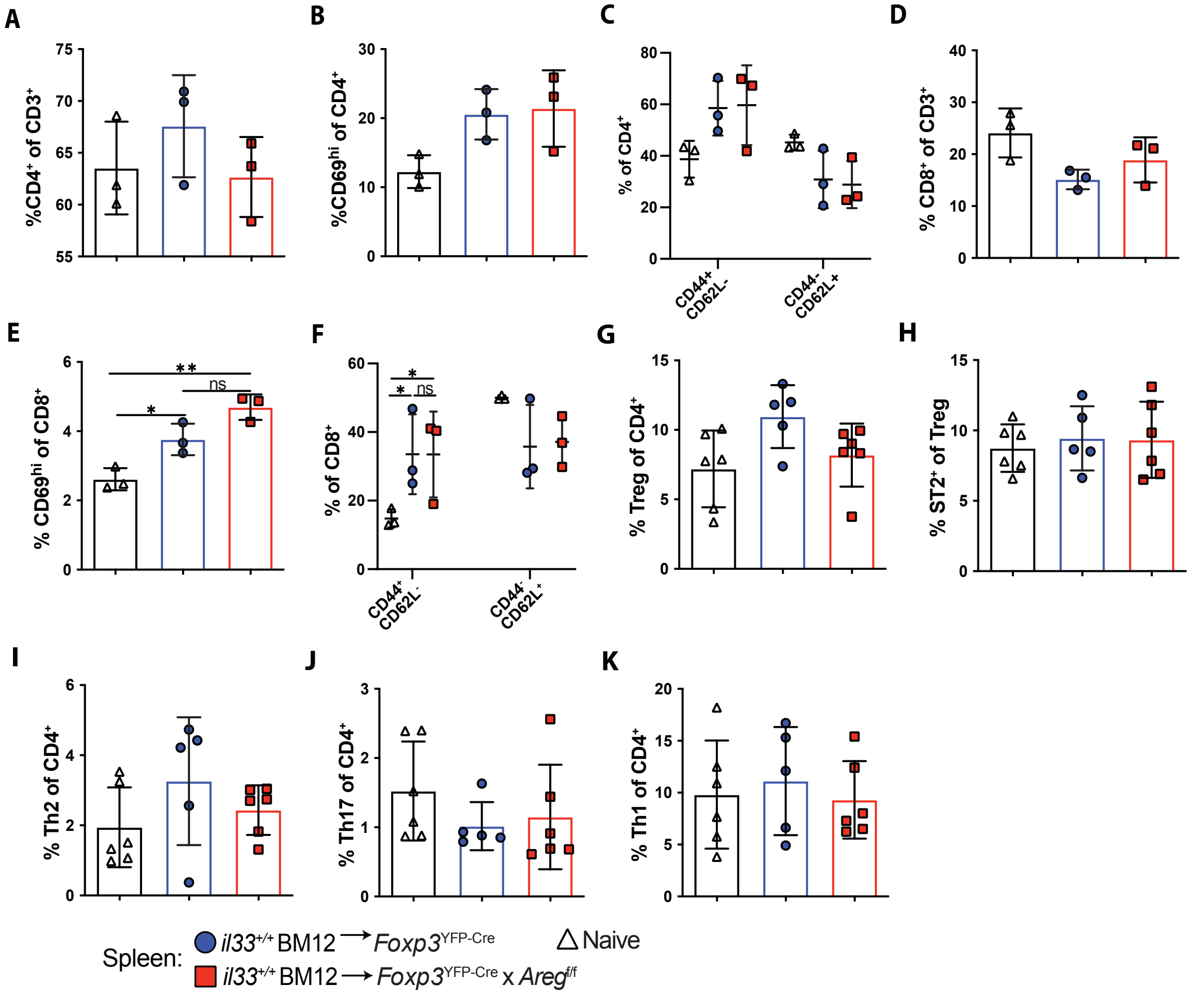

S5. **A lack of Treg-derived Areg does not impact recipient splenic CD4⁺ and CD8⁺ T cell activation, memory, or Th cell skewing**. (**A-K**) Flow cytometry at POD90 of the recipient spleen following donor Bm12 heart grafts transplanted into C57BL/6 *Foxp3*$^{YFP-Cre}$ or *Foxp3*$^{YFP-Cre}$xAreg$^{fl/fl}$ mice. T cells were assessed for activation and memory induction (**A-F**), and Th skewing (**G-K**). n=3-6 grafts/group. Mean±SD. *p≤0.05 by one-way ANOVA with Tukey's multiple comparisons test.

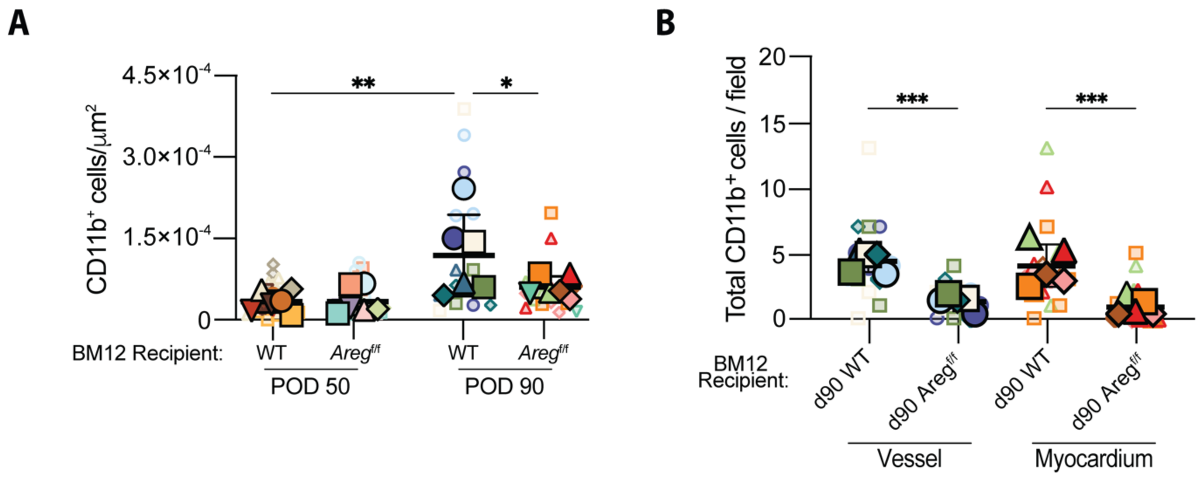

S6. **Myeloid cells are predominately found near the blood vessels and increased by Treg expressed Areg at POD90**. (**A**) CD11b cell quantification of whole sections by IHC at POD50 and POD90. **(B)** CD11b quantification by immunofluorescence of myocardia or vessel areas. n=5-6 grafts/group. Mean±SD. $*p \leq 0.05$; $**p \leq 0.01$; $***p \leq 0.005$ by one-way ANOVA with Tukey's multiple comparisons test.

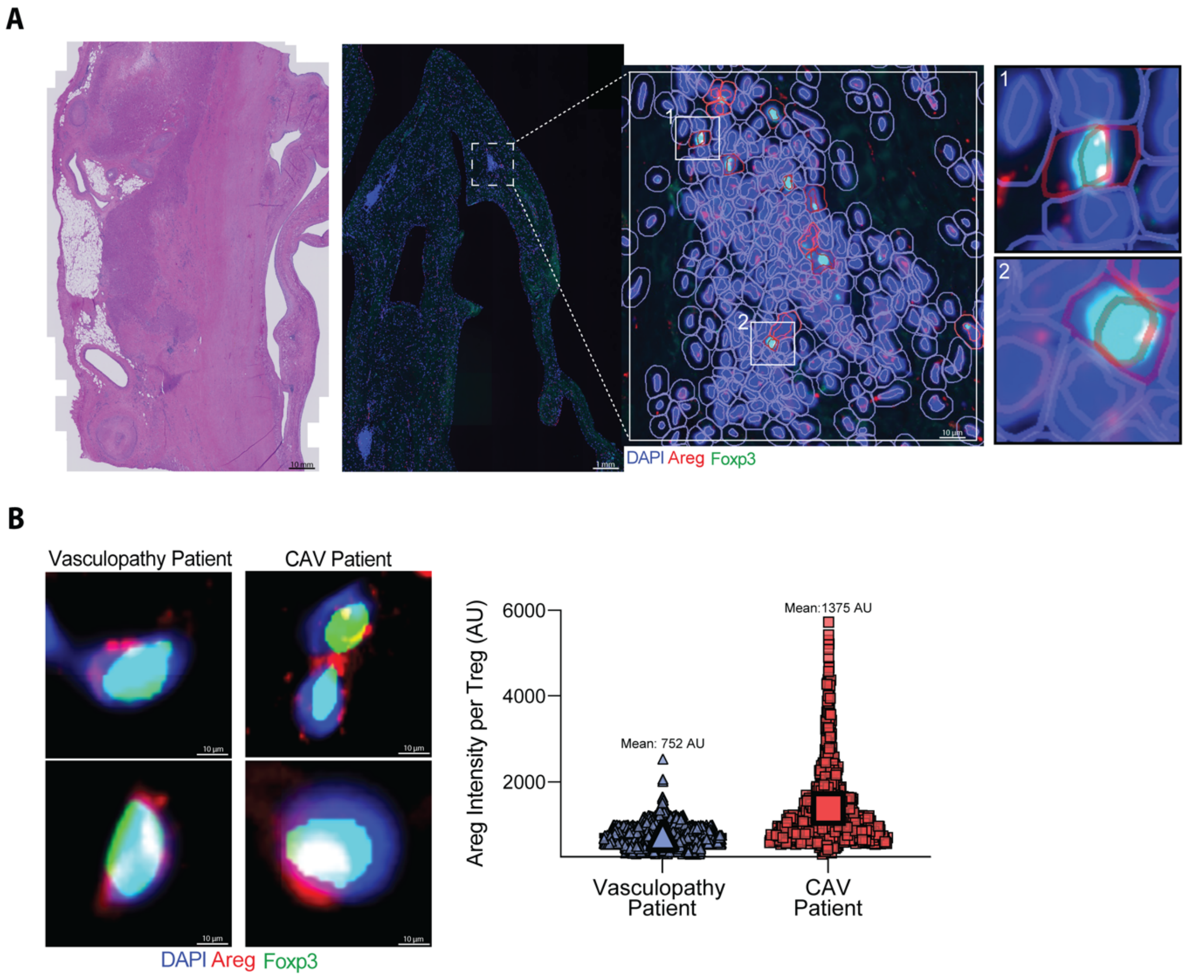

S7. **Areg⁺ Treg are abundant during clinical vasculopathy.** (**A**) Clinical histological analysis overview showing H&E and immunofluorescence with QuPath's trainable cell detector identifying Foxp3⁺ cells. (**B**) Representative Areg staining in Tregs and quantification. Human data are from tissue blocks obtained at the time of re-transplantation from two failing cardiac allografts. n=1 patient/group. Mean±SEM.

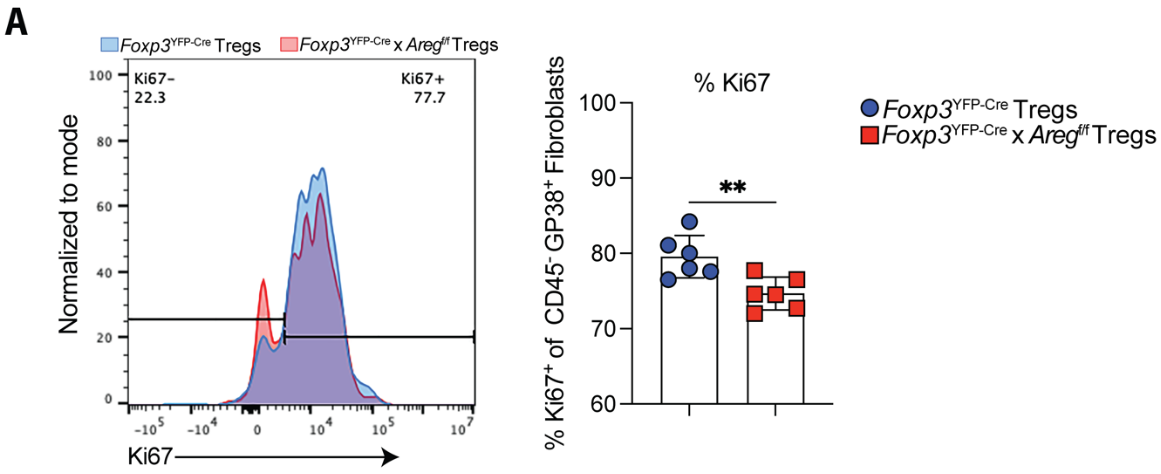

S8. **Treg-derived Areg increases fibroblast proliferation.** (**A**) Quantification of the frequency of Ki67^{+} *St2*$^{-/-}$ fibroblasts co-cultured with FACS sorted Tregs from B6 *Foxp3*$^{\text{YFP-Cre}}$ or *Foxp3*$^{\text{YFP-Cre}}$x*Areg*$^{\text{fl/fl}}$ mice for 72 hours. Mice received 0.5 µg of rmIL-33 via i.p injection for 7 days prior to sorting. Data are pooled from two independent experiments with a total of n=6 wells/group. Student's *t* test **p≤0.01.
